# Climate change causes critical transitions and irreversible alterations of mountain forests

**DOI:** 10.1111/gcb.15118

**Published:** 2020-05-08

**Authors:** Katharina Albrich, Werner Rammer, Rupert Seidl

**Affiliations:** ^1^ Institute of Silviculture University of Natural Resources and Life Sciences (BOKU) Vienna Vienna Austria; ^2^ Ecosystem Dynamics and Forest Management Group Technical University of Munich Freising Germany

**Keywords:** Alps, climate impacts, forest dynamics, forest simulation model, mountain forest landscape, resilience, topographic buffering

## Abstract

Mountain forests are at particular risk of climate change impacts due to their temperature limitation and high exposure to warming. At the same time, their complex topography may help to buffer the effects of climate change and create climate refugia. Whether climate change can lead to critical transitions of mountain forest ecosystems and whether such transitions are reversible remain incompletely understood. We investigated the resilience of forest composition and size structure to climate change, focusing on a mountain forest landscape in the Eastern Alps. Using the individual‐based forest landscape model iLand, we simulated ecosystem responses to a wide range of climatic changes (up to a 6°C increase in mean annual temperature and a 30% reduction in mean annual precipitation), testing for tipping points in vegetation size structure and composition under different topography scenarios. We found that at warming levels above +2°C a threshold was crossed, with the system tipping into an alternative state. The system shifted from a conifer‐dominated landscape characterized by large trees to a landscape dominated by smaller, predominantly broadleaved trees. Topographic complexity moderated climate change impacts, smoothing and delaying the transitions between alternative vegetation states. We subsequently reversed the simulated climate forcing to assess the ability of the landscape to recover from climate change impacts. The forest landscape showed hysteresis, particularly in scenarios with lower precipitation. At the same mean annual temperature, equilibrium vegetation size structure and species composition differed between warming and cooling trajectories. Here we show that even moderate warming corresponding to current policy targets could result in critical transitions of forest ecosystems and highlight the importance of topographic complexity as a buffering agent. Furthermore, our results show that overshooting ambitious climate mitigation targets could be dangerous, as ecological impacts can be irreversible at millennial time scales once a tipping point has been crossed.

## Graphical abstract

Climate change is expected to have strong impacts on mountain forests, changing their size structure and species composition. We used a forest landscape model to simulate climate change impacts on a Central European mountain forest landscape. The forest exhibited extreme changes in structure and composition, shifting to an alternative state under stronger climate forcing. Complex mountain topography helped to buffer the impacts of climate change but changes in forest structure and composition were not easily reversible even after the climate forcing was reversed.

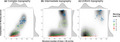

## 1 INTRODUCTION

Recent environmental changes have pushed many ecosystems to the margins of their historic operating space (Duncan, McComb, & Johnson, 2010; Keane, Hessburg, Landres, & Swanson, 2009), increasing the likelihood of abrupt changes in ecosystem characteristics and processes (Scheffer, Carpenter, Foley, Folke, & Walker, 2001). As future changes in the climate system are likely (Good et al., 2011; IPCC, 2013), an important focus of current ecological research is to understand whether ecosystems will respond gradually or abruptly to increasing climate forcing (Turner et al., 2020; van Nes et al., 2016). The growing awareness of tipping points in ecological systems has strongly influenced current targets of climate policy (Schellnhuber, Rahmstorf, & Winkelmann, 2016). Yet, for many systems, it remains unclear whether tipping points exist, and if so, whether limiting climate warming to below +2° is sufficient to prevent critical transitions (i.e., abrupt changes from one ecosystem state to another, Scheffer, 2009). A key question of current ecological research is thus to elucidate how ecosystems respond to increasing levels of warming and quantify the relevant driver–state relationships (Ratajczak et al., 2018).

The concept of resilience provides a powerful framework for studying critical ecosystem transitions in response to environmental change (Johnstone et al., 2016; Ratajczak et al., 2018; Scheffer et al., 2001). Resilience is a broad concept and has been defined in several different ways (Brand & Jax, 2007; Nikinmaa et al., 2020). Here, we focus on ecological resilience, pioneered by Holling (1973) and defined as the ability of “a system to experience shocks while retaining essentially the same function, structure, feedbacks, and therefore identity” (Walker et al., 2006). In this definition, resilience is measured as the amount of perturbation (e.g., change in climate variables) a system can absorb before reaching a tipping point or threshold beyond which it transitions into an alternative state. When a threshold is crossed, systems may also exhibit hysteresis. A hysteretic system will not return to its initial state along the same path even if the driver variable is returned to its pre‐threshold level. This means that the driver variable has to be brought to an even lower level to allow the system to return to its initial state. It may also cause a system to be locked in an alternative, possibly undesirable state despite the removal of the initial forcing. Previous forest research on this question has largely focused on the forest–grassland ecotone and on tropical rainforests (Cowling & Shin, 2006; Good et al., 2011; Levine et al., 2016), finding clear evidence for alternative states and hysteresis (Beckage, Platt, & Gross, 2009; Staal, Dekker, Xu, & Nes, 2016; van Nes, Hirota, Holmgren, & Scheffer, 2014). Tipping points and hysteresis remain understudied for extratropical systems (but see e.g., Hansen, Braziunas, Rammer, Seidl, & Turner, 2018, Miller, Thompson, Tepley, & Anderson‐Teixeira, 2018, e.g., for potential tipping points in North American systems, and Scheffer, Hirota, Holmgren, Nes, & Chapin, 2012 for an investigation of critical transitions in boreal systems), and to our knowledge no investigation of potential critical transitions exists for forest ecosystems in Central Europe to date.

While resilience research has made large conceptual advances in recent years, applying the concept to specific ecosystems has proven difficult, with measuring and quantifying resilience being particularly challenging (Ingrisch & Bahn, 2018; Reyer et al., 2015; Scheffer, Carpenter, Dakos, & Nes, 2015). In long‐lived terrestrial ecosystems such as forests, critical transitions are frequently only apparent years to decades after they have taken place (Hansen et al., 2018; Thrippleton, Bugmann, & Snell, 2018). Furthermore, experimental manipulations—which are an important means to explore resilience to environmental changes (Butitta, Carpenter, Loken, Pace, & Stanley, 2017; Schröder, Persson, & Roos, 2005)—are of limited applicability for studying forest systems at the ecosystem to landscape scale. Simulation models help address these challenges in studying the resilience of forest ecosystems (Egli, Weise, Radchuk, Seppelt, & Grimm, 2018; Reyer et al., 2015; Seidl, Spies, Peterson, Stephens, & Hicke, 2016). They allow the investigation of extended temporal and spatial domains in an efficient manner and can quantify the effect of changes in the environment for which no past analogues exist.

Mountain areas are particularly exposed to climatic changes (Pepin et al., 2015), and life in mountains is strongly temperature limited. This puts mountain ecosystems at particular risk of severe climate change impacts (Palomo, 2017; Thuiller, Lavorel, Araújo, Sykes, & Prentice, 2005), and makes them important study systems for early detection of the potential consequences of climate change (Beniston, 2003). At the same time, mountain ecosystems are characterized by high topographic complexity, which is increasingly recognized as an important factor modulating the impacts of climate change on vegetation (Ashcroft, Chisholm, & French, 2009; Senf & Seidl, 2018). Complex topography may, for example, provide sheltered (e.g., cooler and moister) sites where species can persist even though the general climate becomes unfavorable for them. Such refugia could subsequently be the nuclei of recolonization once environmental conditions return to a more suitable level, overall fostering a more buffered response to climate drivers than in topographically homogenous landscapes (Serra‐Diaz, Scheller, Syphard, & Franklin, 2015). Therefore, we hypothesize that complex topography reduces the probability of threshold responses and fosters resilience of mountain ecosystems (Turner, Donato, & Romme, 2013; van Nes & Scheffer, 2005).

We applied an individual‐based forest simulation model to study the resilience of a mountain forest landscape in the European Alps to changes in temperature and precipitation. Specifically, we focused our analysis on the response of forest size structure and species composition to climate change. Structurally, a defining characteristic of the current mountain forests of the Alps is the presence and number of large trees, while the key species dominating their potential natural as well as current vegetation composition is Norway spruce (*Picea abies* (L.) Karst.). Both the characteristic size structure and species composition are also relevant to locally important ecosystem services such as timber production, protection from natural hazards and carbon storage (Seidl et al., 2019; Tappeiner, Tasser, Leitinger, Cernusca, & Tappeiner, 2008). Here we quantified the resilience of these attributes to climate change, asking (a) whether there are threshold responses in forest composition and size structure to progressive changes in the climate system, (b) how topographic complexity influences the response of the landscape to climate change, and (c) whether climate‐mediated changes in the ecosystem are reversible once the climate forcing is removed. To isolate the role of climate in driving forest change, we analyzed equilibrium size structure and species composition. This allowed us to control for the substantial land‐use legacies that are present in forest ecosystems throughout the Alps (Bebi et al., 2017), and to eliminate transient dynamics in the identification of system attractors (Schröder et al., 2005).

## 2 MATERIALS AND METHODS

### 2.1 Study landscape

**FIGURE 1:**
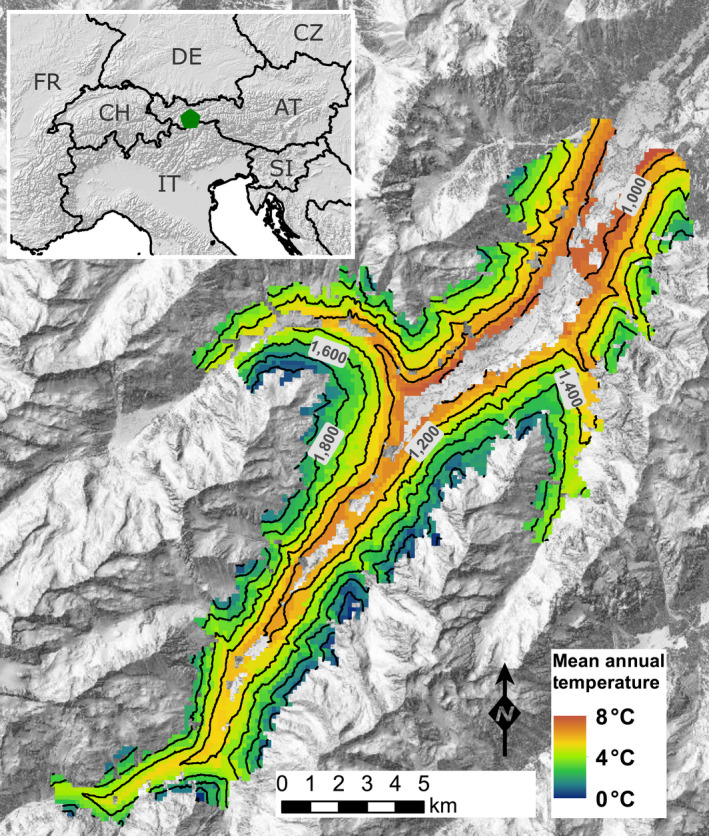
Map of the study area, showing the historical mean annual temperature (1961–2014) and the position of the landscape within Central Europe (insert). Isolines are 100 m apart (Basemaps from basemap.at, copernicus.eu, ec.europa.eu)

The Stubai Valley study landscape is located in the central Alps in Tyrol, Austria (47.10°N, 11.29°E). It is characterized by a strong vertical gradient from 900 m a.s.l. (valley bottom) to the timber line at 2,000 m a.s.l., with the highest mountain peaks exceeding 3,500 m a.s.l. The most important tree species of both the natural and current vegetation are Norway spruce, European larch (*Larix decidua* Mill.), and Swiss stone pine (*Pinus cembra* L.). Mean annual temperature for the period 1961–2014 was 4.1°C, sharply decreasing with elevation (from 7.2°C to 0.6°C, Figure 1). Mean annual precipitation was 998 mm, increasing with elevation (from 826 to 1,163 mm). Historically, the area has been influenced by human land‐use such as forest management, grassland management including cattle grazing on alpine pastures, and tourism (Tappeiner et al., 2008). Here we focused on the area of the Stubai Valley that is currently forested, a contiguous land area of 4,811 ha.

### 2.2 Simulation model

We used iLand, the individual‐based forest landscape and disturbance model (Seidl, Spies, et al., 2012) to simulate forest species composition and size structure under climate change. iLand is a spatially explicit landscape model simulating forest ecosystem dynamics. Detailed descriptions of iLand can be found in Seidl, Rammer, and Spies (2014), Seidl, Rammer, Scheller, and Spies (2012), Seidl, Spies, et al. (2012), Thom, Rammer, and Seidl (2017b), and Thom, Rammer, Dirnböck, et al. (2017); here we focus on describing the core processes of particular relevance for this study. In iLand, the vegetation state is updated annually based on dynamically simulated processes of tree growth, mortality, and regeneration. Productivity is calculated monthly based on a resource use efficiency approach (Landsberg & Waring, 1997) and is contingent on environmental conditions and species traits. Relevant environment variables include climate (temperature, precipitation, radiation, and vapor pressure deficit, all considered at daily resolution) and soil conditions (effective soil depth; sand, silt and clay fractions; and nitrogen availability; all temporally invariant throughout the simulation). Carbohydrate allocation in trees is calculated annually based on allometric ratios and is sensitive to a tree's competitive status. The mortality probability of a tree is influenced both by its carbon balance (stress‐related mortality) and by its size and age (species‐specific life‐history traits). While iLand is also able to simulate tree mortality from natural disturbances and management (Rammer & Seidl, 2015; Seidl & Rammer, 2017; Seidl, Rammer, & Blennow, 2014), we did not include these factors in our study design (see details below). iLand simulates tree regeneration at the grain of 4 m^2^ cells (annual time step), and accounts for the processes of seed dispersal and climate‐dependent establishment, as well as seedling and sapling growth (Seidl, Spies, et al., 2012). The model simulates the ecosystem water cycle dynamically at daily time steps (spatial grain of 1 ha cells), with water availability directly influenced by precipitation, soil properties (soil depth and texture), and the presence and composition of forest vegetation. The model has been applied and evaluated for multiple landscapes in Central Europe (Dobor et al., 2018; Thom, Rammer, Dirnböck, et al., 2017), including the focal landscape of this study (Supplementary Material Figures S1.1–S1.4, Seidl et al., 2019).

### 2.3 Topography scenarios and initial conditions

To investigate the effect of topography on climate responses, we developed three different topography scenarios, hereafter referred to as “complex topography,” “intermediate topography,” and “uniform topography.” The complex topography scenario corresponds to the present topography of the valley with climate and soil properties varying at a grain of 100 m horizontal resolution (see Seidl et al., 2019). Soil input data (soil physical properties, effective soil depth, and plant‐available nitrogen) were based on a map of local forest types and their respective soil conditions (Hotter, Simon, & Vacik, 2013) in combination with measurements from the Austrian Forest Soil Survey (Seidl, Rammer, & Lexer, 2009). Climate data were derived by statistically down‐scaling climate variables from gridded climate data at 1 km resolution, using local weather station data (see Seidl et al., 2019 for details).

While the complex topography scenario represents the high environmental variability present in the landscape, the uniform topography scenario assumes homogeneous soil and climate conditions throughout the landscape. With regard to soil properties, we used the median values of the complex topography scenario (29.7 cm of effective soil depth, i.e., the soil depth after subtracting coarse materials, 67.4 kg/ha of plant‐available nitrogen). We applied the most common combination of sand, silt, and clay fractions to the entire landscape (45% sand, 37.5% silt, and 17.5% clay content). As the driving climate, we assigned a spatially homogeneous climate time series based on the climatology most similar to the landscape mean (temperature = 4.31°C, precipitation = 969 mm, radiation = 10.3 MJ m^−2^ day^−1^, and vapor pressure deficit = 0.254 kPa).

A third, intermediate topography scenario was created by reducing the heterogeneity of the complex topography scenario. For this intermediate scenario, temperature variation in space was rescaled to the 25th and 75th percentiles of the range of the complex topography scenario. Between these rescaled extremes, all pixels were assigned a new climatology following a quantile mapping approach, keeping the gradients of temperature, precipitation, and radiation consistent. Soil variables were aggregated to larger spatial groups to also create intermediate heterogeneity in soils (see Supplementary Material S1 section 2).

Topography not only modulates climate and soil conditions but also influences the dispersal of propagules. We accounted for this effect by assuming different dispersal and migration pathways in the topography scenarios. In addition to seeds from adult trees present on the landscape, forest areas surrounding the landscape can act as seed sources, contributing a small amount of seeds from species not currently present on the landscape (total species pool: 30 central European species, equal immigration probabilities per unit area). In the complex topography scenario, only a small area acts as an external seed source, representing forests adjacent to the study area at the entrance of the valley. This is in line with current conditions, where the influx of seeds occurs mainly from the north and is strongly limited from all other sides by the (partly glaciated) mountain range surrounding the valley. This barrier effect was assumed to be independent of climate scenario. In the uniform topography scenario, new species could migrate into the study area from all sides, representing adjacent forests without natural barriers to seed dispersal. We tested the impact of these two seed source scenarios for the intermediate topography scenario, which was simulated with both seed areas allowing a direct comparison (Supplementary Material Figures S1.5–S1.9). The initial vegetation state for the three topography scenarios—representing the current potential natural vegetation—was derived via spin‐up simulations, running iLand for 1,000 years under historic climate (years 1961–2000, randomly drawn with replacement) in the absence of management.

### 2.4 Study design

To test for tipping points with increasing climate forcing, we simulated a stepwise change in temperature (between +0°C and +6°C), with each temperature interval lasting 1,000 years. The effect of this stepwise temperature change was evaluated under different precipitation scenarios (between −0% and −30% change in mean annual precipitation) to assess the independent effects of precipitation and temperature. Temperature and precipitation changes were chosen to include potential temperature increases and precipitation losses in the region expected under RCP 8.5 by the end of the 21st century (see Seidl et al., 2019). This allowed us to identify under which combined climate forcing (if any) a critical transition occurs. To ensure realistic temporal variation and autocorrelation of climate variables, we used statistically downscaled future climate scenario data (i.e., from the GCM‐RCM combination of HadGEM2‐ES and CLMcom‐CCLM4‐8‐17 driven by RCP 8.5, see also Seidl et al., 2019 for details on how the climate scenario was derived) as basis for our climate scenarios. For each stepwise increase in temperature, we identified periods with a minimum length of 20 years in the downscaled climate scenarios where the simulated temperature change matched the respective target (i.e., +1°C: 2001–2022, +2°C: 2016–2046, +3°C: 2036–2067, +4°C: 2055–2075, +5°C: 2061–2091, +6°C: 2079–2099), while climate for the +0°C level was sampled from historical records (1961–2000). We randomly sampled 1,000 years with replacement from these periods to generate stepwise changes in climate. We rescaled precipitation to match the historical mean of the baseline period (1951–2000) while conserving interannual precipitation patterns. We then created four different precipitation change scenarios, corresponding to historical mean annual precipitation and −10%, −20%, and −30% relative to historical conditions. These changes in precipitation correspond to the climate model data used for extracting temperature changes (HadGEM2‐ES and CLMcom‐CCLM4‐8‐17 driven by RCP 8.5), ensuring consistency between variables in the generically constructed climate scenarios. We note, however, that a wide variety of precipitation changes are projected for the future in our study area by different climate models (see also Seidl et al., 2019). Climate varied spatially at 100 m resolution based on the underlying topography (see also Section 2.3 above). A single climate series was used for all simulated cells in the uniform scenario, while the climate varied between cells in the complex and (with reduced level of variation) the intermediate scenarios.

To address our research question regarding the reversibility of climate impacts and test for possible hysteresis, we first simulated a stepwise increase in temperature up to +6°C (which is the expected temperature increase in our study landscape by the end of the 21st century under RCP 8.5, Seidl et al., 2019), followed by a symmetrical stepwise decrease in temperature. This sequence of temperature change was simulated for each of the above‐described precipitation change scenarios, with precipitation remaining at the same level throughout the respective simulations. We also tested an earlier reversal of the temperature forcing, at warming levels of +4°C (see Supplementary Material Figures S1.10–S1.13 for details). As we were interested in climate‐mediated changes in the natural vegetation composition, each change step was simulated for 1,000 years, allowing the system to find a new dynamic equilibrium with climate (see Supplementary Material Figure S1.24 for a conceptual drawing). We evaluated the development of biomass and species composition over time (see also Thom, Rammer, & Seidl, 2017a) and found that a simulation duration of 1,000 years per temperature step was sufficient for the system to obtain a dynamic equilibrium with climate. A doubling of the simulation time did not yield significantly different results (Supplementary Material Figures S1.14–S1.19 but note that larger temperature increments would require longer equilibration times, see Supplementary Material Figures S1.20–S1.23). In all, 10 replicated simulations covering the full 13,000‐year sequence of warming and cooling were run for each combination of topography and precipitation to account for stochasticity in the model (e.g., from mortality and regeneration processes).

### 2.5 Analysis

We analyzed the resilience of forest size structure and species composition (“of what”) to changes in the climate system (“to what”). The forests of our study landscape are currently characterized by a strong dominance of Norway spruce, a species that is important throughout the mountain forests of the Alps (Mayer, 1984). Consequently, we chose the share of Norway spruce (in percent of total basal area) as our focal indicator for forest composition, asking whether this defining species of current mountain forests will still play a dominant role in the late‐seral forests emerging under climate change. Current mountain forests in the Alps also have relatively high number of large diameter trees (Bebi et al., 2017). Large trees are important for both biodiversity (Franklin et al., 2002) and ecosystem service provisioning (e.g., in the context of protecting settlements from gravitational natural hazards, where a sufficient number of large trees is needed to fulfill at protective function; Moos et al., 2018). We asked whether this characteristic feature (here quantified as the number of trees per hectare with a diameter at breast height of >30 cm) could be retained under future climate. We assessed the robustness of our findings to different indicator formulations by conducting analyses for alternative diameter thresholds and a broader species portfolio (see Supplementary Material Figures S1.25 and S1.26). We analyzed both indicators at the landscape level, averaging simulation results for the last 50 years of each 1,000‐year climate period.

We visually analyzed the two resilience indicators both separately and in combination for tipping points and hysteresis effects after switching from warming to cooling trajectories. A tipping point was defined as a nonlinear change with increasing climate forcing. We identified hysteresis if the simulated system paths for the same climate forcing differed between warming (+0°C to +6°C) and cooling trajectories (+6°C to +0°) of the simulation. To quantify differences in the full species composition and diameter distribution beyond the two focal indicators (number of large trees, Norway spruce share), we calculated the Bray–Curtis Dissimilarity (Bray & Curtis, 1957) between warming and cooling trajectories at the end of each climate period. This index allows for the analysis of differences in species composition between groups and can be calculated both from counts of individuals and from proportions. An index of 0 indicates perfect similarity between two groups (here: the warming and cooling trajectories of the system) while an index of 1 means no overlap. We used the Bray–Curtis Dissimilarity for both species composition and stand size structure, interpreting the number of individuals per 10 cm diameter class similarly to the number of individuals per species. The differences across species and diameter distributions were tested for significance using a PERMANOVA approach. All analyses were done using version 3.5.1 of the R statistical computing language (R Core Team, 2019), in particular applying the packages tidyverse (Wickham, 2017), RSqlite (Müller, Wickham, James, & Falcon, 2018) and vegan (for PERMANOVA and the Bray–Curtis Dissimilarity, Oksanen et al., 2018).

## 3 RESULTS

### 3.1 Forest tipping points with climate warming

**FIGURE 2:**
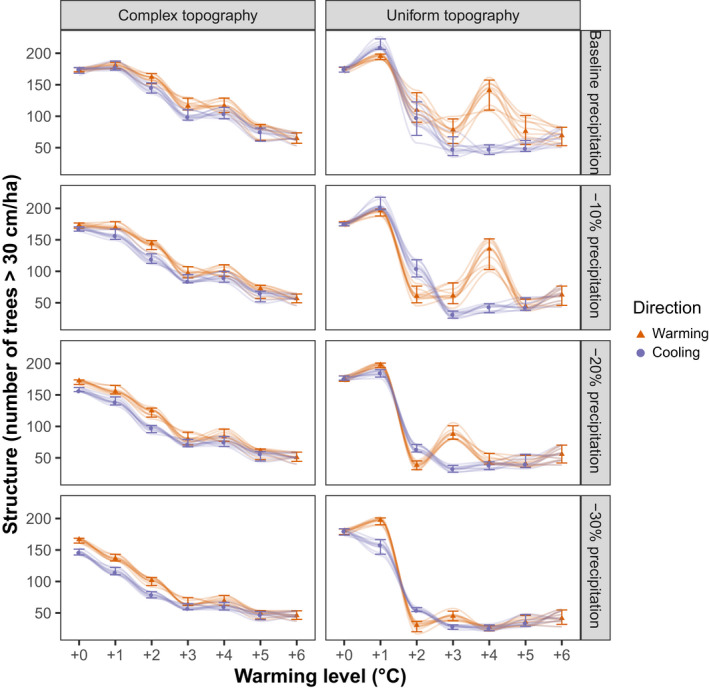
The response of forest size structure (here described as the number of trees > 30 cm in diameter) to climate warming (red, triangles) and subsequent cooling (purple, circles). Values describe the state of the landscape at equilibrium (median, 5th and 95th percentiles across 10 replicates) and trajectories for all simulated replicates are shown. Trajectory lines are fitted using a LOESS model

Climate change strongly influenced forest size structure (Figure 2; Supplementary Material Figure S1.27). Topography distinctly modulated the shape of this response. In the complex topography scenario, climate impacts were buffered, and the number of large trees decreased gradually with increasing temperatures, dropping from around 175 trees > 30 cm dbh/ha under current climate to around 50 trees per hectare under the +6°C scenario. In contrast, we found a distinct tipping point in the simulated number of large trees in the uniform topography scenario, with a pronounced shift between warming levels of +1°C and +2°C. The uniform topography scenario resulted in three attractors for forest size structure, with a local optimum at +3°C warming, resulting from a dominance of European beech at this particular warming level. This local optimum shifted to higher warming levels in scenarios with higher water availability (Figure 2). The intermediate topography scenario showed similar behavior with stem numbers reaching their maximum at +2°C, a local optimum at +4°C (regardless of precipitation), and a minimum at +6°C (Supplementary Material Figure S1.5). Overall, the changes from one temperature step to the next in this scenario were more gradual than in the uniform scenario, but less linear than in the complex scenario. More broadly, the number of large trees was reduced under climate change, while the number of smaller trees (especially in diameter classes below 20 cm) strongly increased, resulting in a higher overall stem density under climate change (Supplementary Material Figures S1.7 and S1.27).

**FIGURE 3:**
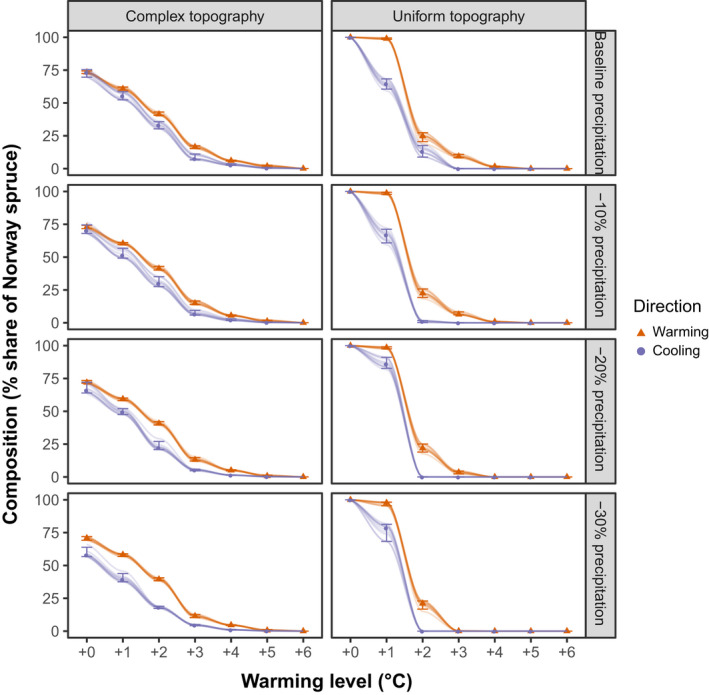
The response of forest composition (here described as the share of Norway spruce on total basal area) to climate warming (red, triangles) and subsequent cooling (purple, circles). Values describe the state of the landscape at equilibrium (median, 5th and 95th percentiles across 10 replicates) and trajectories for all simulated replicates are shown. Trajectory lines are fitted using a LOESS model

Forest composition also changed with climate warming. The basal area share of Norway spruce decreased sharply, with the species being virtually absent from the landscape at warming levels of >5°C (Figure 3; Supplementary Material Figures S1.8 and S1.28). Spruce was initially outcompeted by beech which—at even higher levels of warming—was succeeded by oak (*Quercus robur* L., *Quercus petraea* (Matt.) Liebl.) and Scots pine (*Pinus sylvestris* L.; see also Supplementary Material Figure S1.28). We observed a threshold response under uniform topography, with a 75% decrease in spruce share at +2°C and almost complete extirpation at +4°C. Spruce share declined gradually with increasing warming in the complex topography scenario. In the intermediate topography scenario, spruce decline was intermediate given sufficient external seed sources (large seed area, Supplementary Material Figure S1.6). While it had a more noticeable tipping point in spruce share than the complex scenario, this tipping occurred at higher temperatures than in the uniform scenario (at +3°C of warming, regardless of precipitation and seed availability scenario). Limiting the influx of seeds to the entrance of the valley (small seed area) strongly increased the variability between simulated replicates in the intermediate topography scenario.

The strong elevation gradients in the complex topography scenario created climate refugia for spruce on the landscape (Supplementary Material Figure S1.30). Overall species change was strong across the whole elevation range, with oaks occuring even at the highest elevation (>2,000 m a.s.l.) under +6°C. However, individual spruce trees were able to persist in the highest reaches of the landscape even under the hottest and driest scenarios (cf. the maps in the Supplementary Material S2). At the same time, *P. cembra*, which is the species forming the timber line in the landscape currently, was lost completely at warming levels of above +2°C, and was not able to return after the climate forcing was reversed (Supplementary Material Figure S1.30).

### 3.2 Hysteresis between warming and cooling trajectories

Equilibrium vegetation structure and composition differed between the simulated warming and cooling trajectories, indicating a strong hysteresis effect (Figures 2 and 3). Hysteresis effects were generally stronger under uniform topography compared to complex topography for both indicators. For example, under uniform topography forest size structure exhibited a local optimum at +3°C under warming but not cooling trajectories. The hysteresis effect was stronger for forest species composition than for forest size structure. Under uniform topography, spruce shares remained low in the cooling trajectories until recovering dominance at +1°C. In contrast, spruce share increased gradually and recovery started at higher temperatures during cooling trajectories when topography was complex. This was despite the limiting effect of external seed availability (i.e., seeds of trees not currently present on the landscape entering the simulation only in a limited area at the entrace of the valley) in the complex topography scenario which generally increased hysteresis in species composition (see intermediate topography scenario, Supplementary Material Figure S1.6).

Quantitative analyses across the full species and diameter distribution supported findings from visual analysis of simulation trajectories (Tables 1 and 2). Bray–Curtis Dissimilarity between warming and cooling trajectories was generally lower in the complex topography scenarios across all temperature and precipitation forcings, indicating that uniform topography amplifies hysteresis effects. For forest size structure, the highest dissimilarity occurred at +1°C regardless of precipitation scenarios when topography was uniform. However, when topography was complex, maximum dissimilarity occurred at +2°C under wetter scenarios (−0% and −10% mean precipitation) and at +0°C under drier scenarios (−20% and −30% mean precipitation). These differences in forest size structure were statistically significant at +2°C for all combinations of topography and precipitation scenarios (Table 1). For forest composition, the biggest differences between warming and cooling trajectories occurred at higher warming levels (between +2°C and +4°C) and depended more strongly on the precipitation scenario simulated. With decreasing precipitation, the temperature of the highest dissimilarity decreased, from +4°C at baseline precipitation to +2°C when precipitation was reduced by 30% (Table 2). Under uniform topography, all simulations returned to their starting point when the temperature forcing was removed completely. Under the complex topography scenario, however, decreasing precipitation resulted in distinctly different size structure and composition of the vegetation even after returning to past temperatures (+0°C forcing level).

**TABLE 1:** Bray–Curtis Dissimilarity quantifying the difference in forest size structure between warming and cooling trajectories at each temperature step separately for each topography and precipitation scenario. A significant difference indicates the presence of a hysteresis effect. The significance of the differences at each step was tested using a PERMANOVA

Temperature change	Precipitation scenario							
	Baseline		Minus 10%		Minus 20%		Minus 30%	
	Topography scenario							
	Complex	Uniform	Complex	Uniform	Complex	Uniform	Complex	Uniform
0	0.010	0.003	0.038**	0.009	0.078***	0.003	0.117***	0.011
1	0.019	0.366***	0.058***	0.352***	0.066***	0.240***	0.078***	0.376***
2	0.058***	0.053**	0.060***	0.124***	0.059***	0.119***	0.058***	0.135***
3	0.032*	0.221***	0.022*	0.138***	0.025	0.090***	0.017	0.034*
4	0.022*	0.293***	0.025*	0.168***	0.033*	0.042**	0.030*	0.004
5	0.014	0.032	0.017	0.010	0.008	0.008	0.003	0.004

Significance levels

* *p* < .05,

** *p* < .01,

*** *p*<=0.001.

**TABLE 2:** Bray–Curtis Dissimilarity quantifying the difference in forest species composition between warming and cooling trajectories at each temperature step separately for each topography and precipitation scenario. A significant difference indicates the presence of a hysteresis effect. The significance of the differences at each step was tested using a PERMANOVA

Temperature change	Precipitation scenario							
	Baseline		Minus 10%		Minus 20%		Minus 30%	
	Topography scenario							
	Complex	Uniform	Complex	Uniform	Complex	Uniform	Complex	Uniform
0	0.051	0.011	0.092	0.012	0.181*	0.011	0.287***	0.012
1	0.089	0.371***	0.184*	0.363***	0.272**	0.150*	0.356**	0.275**
2	0.161*	0.187*	0.275***	0.266**	0.375***	0.740***	0.374***	0.840***
3	0.303***	0.704***	0.327**	0.824***	0.302**	0.656***	0.209*	0.235*
4	0.316**	0.875***	0.298**	0.792***	0.229*	0.192	0.163	0.022
5	0.243*	0.385**	0.177	0.089	0.106	0.022	0.071	0.024

Significance levels

* *p* < .05,

** *p* < .01,

*** *p*<=0.001.

### 3.3 Ecological resilience to climate warming

**FIGURE 4:**
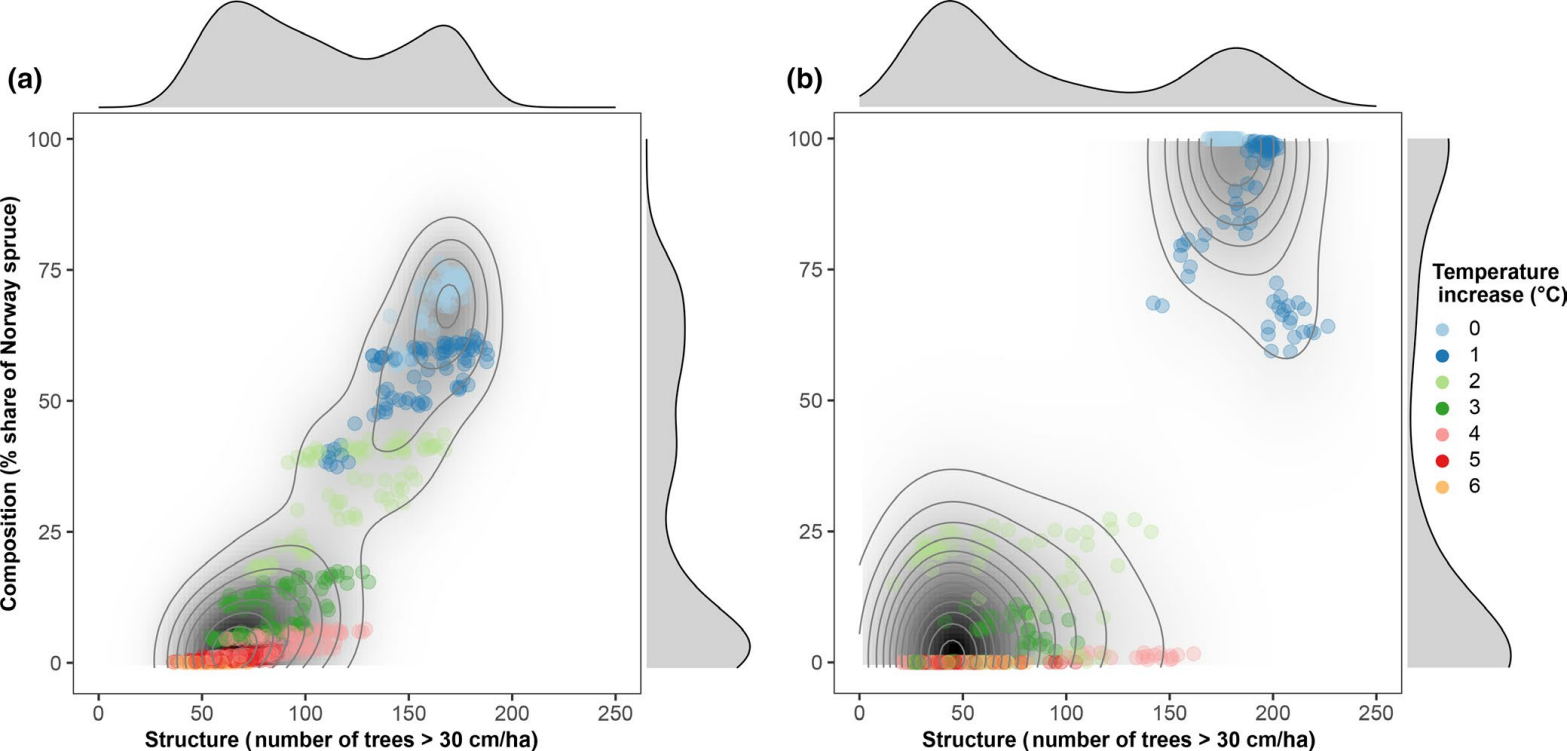
Location of the forest landscape in structure–composition attractor space for different warming levels and the complex (a) and uniform (b) topography scenarios over all precipitation scenarios. Marginal plots and isolines give the probability density of all simulated cases, and indicate two alternative stable states for our study landscape

The attractor landscape emerging from the joint analysis of forest size structure and species composition showed two distinct basins of attraction (Figure 4). Climate change caused a critical transition between the two attractors. Specifically, a warming of +2°C triggered a transition from the current attractor, characterized by a high dominance of Norway spruce and a high number of trees >30 cm in diameter, to an alternative steady state of little to no Norway spruce and considerably smaller sized trees. Topographic complexity reduced the distance between the two basins of attraction. The intermediate topography scenario showed signs of a third attractor at low warming levels due to differences in forest composition (Supplementary Material Figure S1.9). However, the critical transition at a warming level of +2°C occurred regardless of topographic complexity.

## 4 DISCUSSION

### 4.1 Forest response to climate change

Climate change has the potential to profoundly alter forest ecosystems. Here we found evidence for substantial shifts in equilibrium forest composition and size structure under climate change for our study system in the European Alps. The response to increasing levels of warming was strongly nonlinear especially in the absence of steep topographic gradients (uniform scenario). Without the buffering effect of topographic complexity, critical transitions occurred even at weak climate forcings of between +1°C and +2°C. Beyond a warming of between +2° and +3°C relative to historic climate, critical transitions of forest composition and size structure occurred in all simulated scenarios. Reductions in precipitation exacerbated this effect, with critical transitions occurring at lower levels of warming, particularly for the forest size structure indicator investigated here. Critical transitions caused the system—currently characterized by a dominance of conifers and the prevalence of many large trees—to change to an alternative stable state with fundamentally different characteristics, namely a broadleaved‐dominated system characterized by smaller trees. The alternative state emerging from our simulations is a realistic possibility, as forests dominated by oaks (and pines) of smaller dimensions are the dominant forest types in warm and dry valleys of the Southern Alps (Rigling et al., 2013). However, our simulations did not result in a transition to non‐forest, despite simulating warming levels of up to +6°C. Even under the most extreme climate forcings, no more than 2% of the current forest area lost its tree cover after 1,000 simulation years. In contrast to other systems (Enright, Fontaine, Bowman, Bradstock, & Williams, 2015; Hansen et al., 2018; Stevens‐Rumann et al., 2018; Tepley, Thompson, Epstein, & Anderson‐Teixeira, 2017), large‐scale forest loss due to climate change appears unlikely in our study system (but see the following section for methodological limitations).

Topographic complexity buffered the response to climate warming and delayed a landscape‐scale transition of forest size structure and species composition. Our results underline that complex topography and spatial heterogeneity contribute to ecological resilience, which is in line with findings from other systems (e.g., Adams, Barnard, & Loomis, 2014, van Nes & Scheffer, 2005; Virah‐Sawmy, Gillson, & Willis, 2009). Complex topography supports ecological resilience by decoupling the local conditions from the large‐scale average (Daly, Conklin, & Unsworth, 2001), thus providing climate refugia for species (Keppel et al., 2012; Serra‐Diaz et al., 2015). We observed topographically mediated refugia in our simulations, with Norway spruce persisting under higher climate forcings in higher elevations and on north‐facing slopes (see also Supplementary Material Figure S1.30 and maps in Supplementary Material S2). However, the buffering capacity of topography was limited: as temperature change became more extreme (i.e., beyond +3°C), all simulations transitioned to an alternative warm‐adapted stable state regardless of topography and precipitation. Furthermore, complex mountain topography can also have negative effects on major processes of resilience, such as the ability to colonize potential habitat. Mountainous topography, where large areas between forested valleys consist of mountain peaks and glaciers above the timber line, can act as barriers for seed dispersal (Rupp, Chapin, & Starfield, 2001) and thus decrease the adaptive capacity of forests. This could make non‐forest states more likely, especially if warm‐adapted species are not available to colonize the landscape and replace species lost through climate change.

Our results highlight that climate warming above critical thresholds can have irreversible impacts on forest ecosystems at millennial time scales. We identified hysteresis in driver–state relationships, with forest size structure and species composition differing between warming and cooling trajectories. This is—to our knowledge—the first documentation of hysteresis effects in the response of forest ecosystems to climate warming (but see e.g., Staal et al., 2018; van Nes et al., 2014, e.g., of hysteresis responses to changing levels of precipitation). The irreversible climate impacts found here are particularly noteworthy as they persist even after 1,000 years of simulated forest dynamics under a given level of climate change, while previous analyses found that mountain forests in the Alps reach a new equilibrium with climate after roughly 500 years (Thom, Rammer, Dirnböck, et al., 2017; Thom et al., 2017a). The main processes resulting in irreversible climate effects in our simulations are founder effects (Grime, 1998), with returning cool‐adapted specialist species not being able to regain their previous dominance once warm‐adapted generalists have taken hold of important parts of the landscape. Species can disappear quickly from an area once the prevailing environmental conditions exceed their fundamental niche, yet it can take them a long time to recolonize these areas via seed dispersal (Meier, Lischke, Schmatz, & Zimmermann, 2012), particularly if dispersal is limited by topography. As both founder effects and dispersal limitation are amplified by complex topography, the complex scenario showed higher levels of irreversibility after returning the temperature forcing to zero compared to the uniform scenario. This suggests that while complex topography can buffer climate impacts, it is also harder to return to previous system states in mountain areas once species have been lost.

In conjunction with founder effects, dispersal limitations can result in species remaining effectively locked out of areas they previously occupied even though the climate conditions have again returned to suitable levels. This “legacy lock” (Johnstone, Hollingsworth, Hollingsworth, Chaping, & Mack, 2010) is only broken once climate conditions return to levels where the previously dominant species regains its competitive advantage. For example, the areas that are dominated by oak under high levels of climate change are initially taken over by pioneer species (particularly Scots pine, *P. sylvestris* L.) once the climate cools and exceeds the temperature niche of oak. These pioneers have a wide physiological amplitude, which allows them to persist on the landscape at all levels of warming (see also Supplementary Material Table S1.1). Norway spruce, the previously dominating species, only slowly reinvades these areas after being almost completely absent from the landscape under extreme levels of warming (except for small refugia in high elevations in the complex topography scenario). The hysteresis effect for forest size structure is linked to the same processes, as the cooling trajectory has higher shares of pioneer species which do not reach the same dimensions as the spruce‐dominated vegetation types of the warming trajectory.

### 4.2 Methodological considerations

Forest resilience is influenced by complex processes and interactions across temporal as well as spatial domains. Capturing these processes poses a challenge for simulation modeling. iLand is a detailed forest landscape model implementing a high degree of process understanding, yet some processes of potential relevance for forest resilience are incompletely represented in the model. One important example pertains to soil processes: Soil depth and texture are time‐invariant in our simulations, ignoring processes such as soil loss through erosion and changes in soil structure, which could have a lasting impact on forest dynamics (Johnston & Crossley, 2002; Johnstone, Chapin, et al., 2010). Furthermore, nutrient feedbacks between vegetation and soil were not dynamically considered in our simulations. We also did not account for the competitive effect of grasses and herbs, which have the potential to interfere with tree regeneration and therefore change forest development pathways (Thrippleton, Bugmann, Kramer‐Priewasser, & Snell, 2016). Processes such as soil erosion, accelerated decomposition, and increased resource competition from forest floor vegetation all act to amplify climate change impacts (rather than dampen them). Therefore, our quantification of critical transitions and irreversibility are conservative estimates of the expected effects of climate warming.

In our study, we focused on the responses of forest ecosystems to changes in temperature and precipitation, two important drivers of forest dynamics. However, processes such as natural disturbances (wind, bark beetles, wildfire) and human land‐use decisions also influence forest dynamics and resilience. Natural disturbances can enhance forest resilience by fostering response diversity (Dell et al., 2019) but changing natural disturbance regimes could also disrupt forest recovery and therefore reduce resilience (Hansen et al., 2018; Turner, Braziunas, Hansen, & Harvey, 2019). There is a high degree of uncertainty in projections of future disturbance regimes and disturbance interactions as climate changes. In general, disturbances are expected to be an increasingly important factor affecting forests (Lindner et al., 2010; Seidl et al., 2017). For our study landscape, natural disturbances are expected to increase in the coming decades (Seidl et al., 2019). Future efforts should thus assess whether increasing natural disturbances further challenge the climate resilience of our landscape (Enright et al., 2015) or increase its adaptive capacity and therefore decrease hysteresis (Thom, Rammer, Dirnböck, et al., 2017; Thom et al., 2017a).

Rising atmospheric CO_2_ concentration can also influence future forest demographics. In the case of our landscape, this could enhance growth and therefore counteract the effects of increased resource limitation from decreased precipitation (Swann, Hoffman, Koven, & Randerson, 2016; Walker et al., 2019). The persistence of such a CO_2_ fertilization effect, however, remains uncertain (Reyer et al., 2014).

Finally, large parts of our analysis focused on two indicators chosen to represent the size structure and species composition of our study system. While these indicators are well suited to capture defining characteristics of typical mountain forest ecosystems of the Alps, a broader set of indicators could have shown a more nuanced picture of forest responses to climate change. In the case of trees species composition, an analysis at the species level is insightful, as it reveals multiple transitions between forest types, from a landscape‐dominated by spruce to a beech‐dominated system, which is succeeded by oak and pine under extreme climate forcing (Supplementary Material Figure S1.28). More detailed analyses of changes (cf. Supplementary Material Figures S1.29 and S1.30 and Supplementary Material S2) can enhance understanding of the impacts of climate change on ecosystem functioning (Mori, Lertzman, & Gustafsson, 2017; Sakschewski et al., 2016), but were beyond the focus of the current analysis. Furthermore, defining thresholds and transitions in forests is difficult because it inter alia depends on the temporal reference frame applied (see e.g., Thrippleton et al., 2018). Here we addressed this issue by reporting climate change effects on equilibrium forest size structure and species composition, which is less sensitive to the time frame of analysis than transient forest dynamics (Schröder et al., 2005).

### 4.3 Implications

We show that critical transitions of ecosystems can occur already at warming levels of around +2°C (see also Elkin et al., 2013). This suggests that even if the current political climate targets are met, fundamental changes in the characteristics of important forest ecosystems of the Alps are likely. Changes of the magnitude required for causing critical transitions in our study system are expected to occur until the end of this century even under the most optimistic current climate projections (IPCC, 2013). However, we found that topographical complexity can buffer against climate change impacts and allow for smoother transitions to an alternative stable state. Conversely, this means that regions with low topographical complexity (e.g., large regions in the boreal biome, Scheffer et al., 2012) may be particularly at risk of critical transitions under climate change, as evidenced in out intermediate and uniform topography scenarios. This implies that measures adapting to expected climate change impacts are of paramount importance (Halofsky et al., 2017; Keenan, 2015; Messier et al., 2015; Millar, Stephenson, & Stephens, 2007; Seidl, Rammer, & Lexer, 2011). We also found that climate warming was irreversible on millennial time scales under some scenarios. Given the gap between targets of current climate policy (aiming to limit anthropogenic warming to below +2°C/+1.5°C, UNFCCC, 2015) and actual greenhouse gas emissions, a temporal exceedance of the political target (“overshoot”) is likely (Geden & Löschel, 2017; Ricke, Millar, & MacMartin, 2017). While such an overshoot corridor would increase political flexibility in reaching the targets agreed in Paris, our findings show that it could have lasting effects on ecosystems. The nonlinearity and irreversibility of climate impacts demonstrated here thus call for timely and effective action to mitigate climate change.

## Supporting information

supinfo S1

supinfo S2

## Data Availability

The data that support the findings of this study are openly available on Figshare at http://doi.org/10.6084/m9.figshare.12091935. Technical model documentation of iLand as well as the executable and model source code are available online at www.iland.boku.ac.at.
