# Supplementary material for: Climate change causes critical transitions and irreversible alterations of mountain forests
Source: Glob Chang Biol. 2020 May 8;26(7):4013–27. doi: 10.1111/gcb.15118 (PMC7317840; doi:10.1111/gcb.15118)
Supplement: Supplementary file 1 — supinfo S1 [file GCB-26-4013-s001.docx]

Supplementary Material S1 for:

**Climate change causes critical transitions and irreversible alterations of mountain forests**

Katharina Albrich, Werner Rammer, Rupert Seidl

**1. Evaluation of model performance for the Stubai Valley.**

The model was tested in depth regarding its ability to reproduce vegetation patterns following the pattern-oriented modelling approach by Grimm et al. (2005). Specifically, we compared model output to inventory data, local yield tables and vegetation maps for the study area. We here present selected results of the evaluation, relevant for the study at hand. For the full set of evaluations performed for the Stubai valley we refer to Seidl et al. (2019, supplementary Material).

| 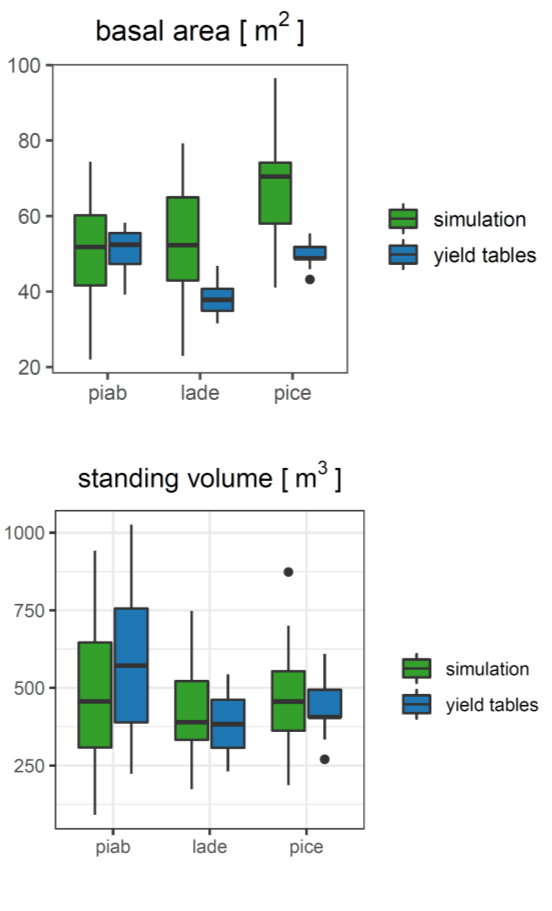 | 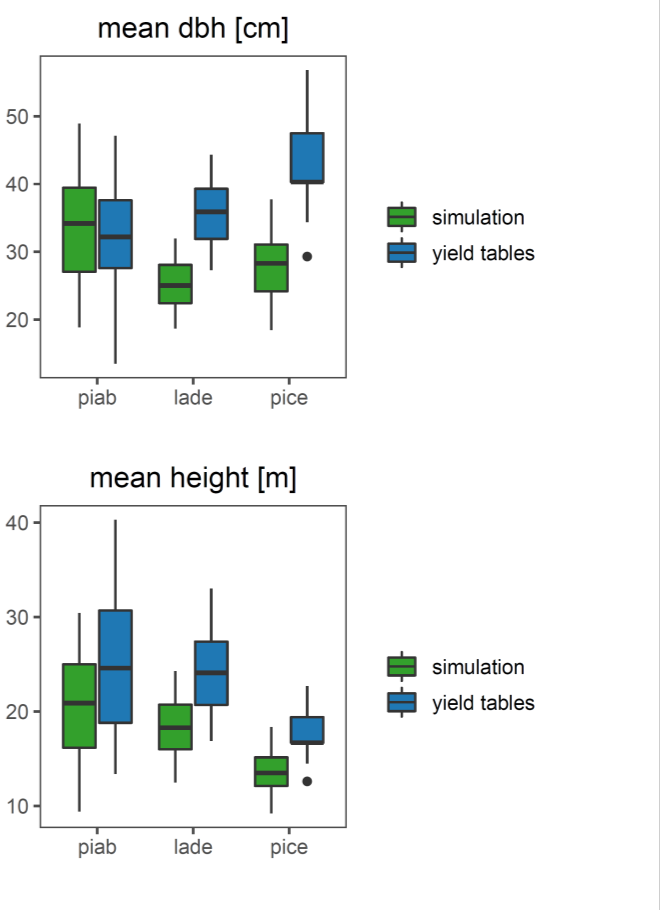 |
| --- | --- |

**Figure S1.1:** Comparison of simulated stand basal area (m^2^ ha^-1^, top left), mean stand dbh (cm, top right), standing volume (m^3^ ha^-1^, bottom left), and mean stand height (m, bottom right) to reference values from yield tables after 70 years of simulation. n= 150 of randomly selected stands distributed throughout the study landscape. Piab= Norway spruce, Lade= European larch, Pice= Swiss stone pine (from Seidl et al. 2019).


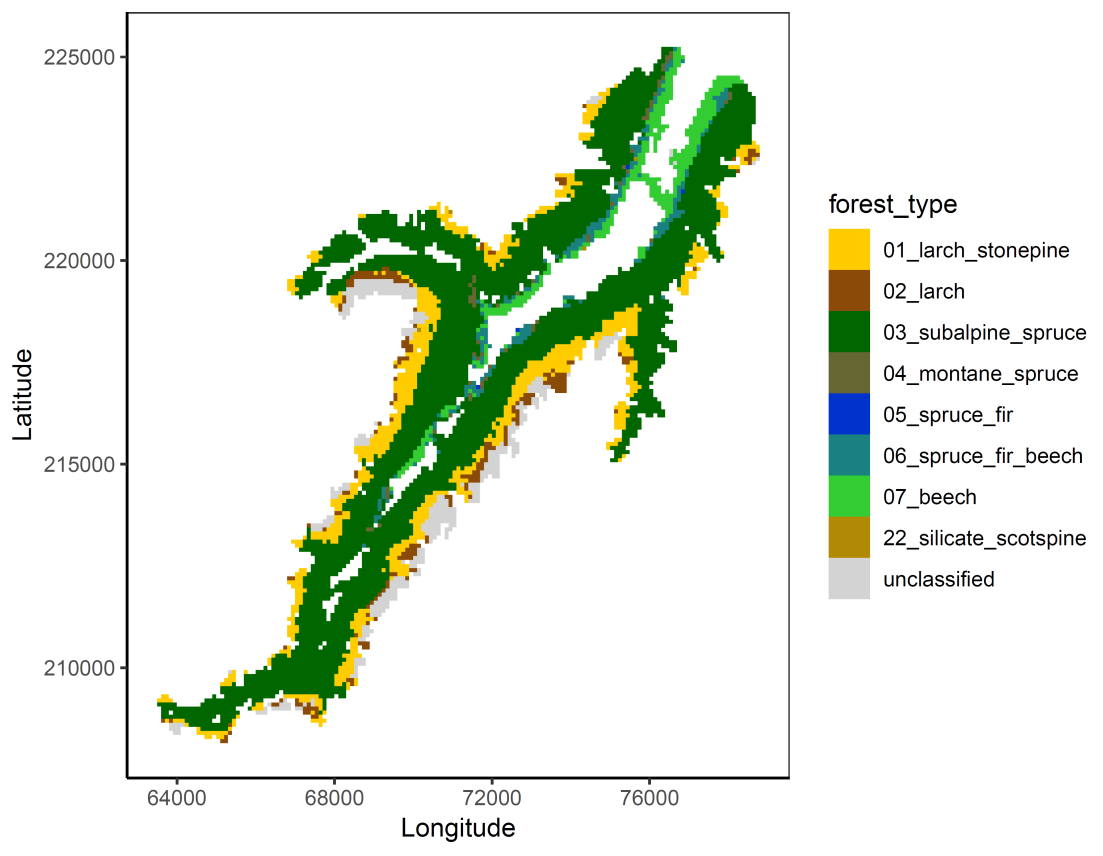


**Figure S1.2:** Simulated forest types of the potential natural vegetation (from Seidl et al. 2019).


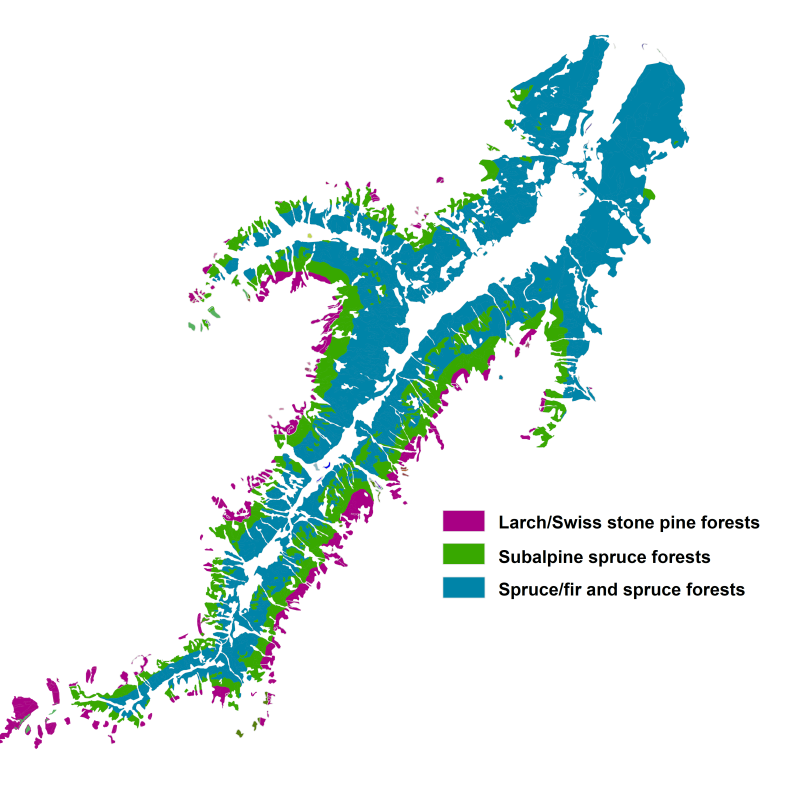


**Figure S1.3:** Reference forest types from the local forest type classification (Hotter et al. 2013, Seidl et al. 2019)

**
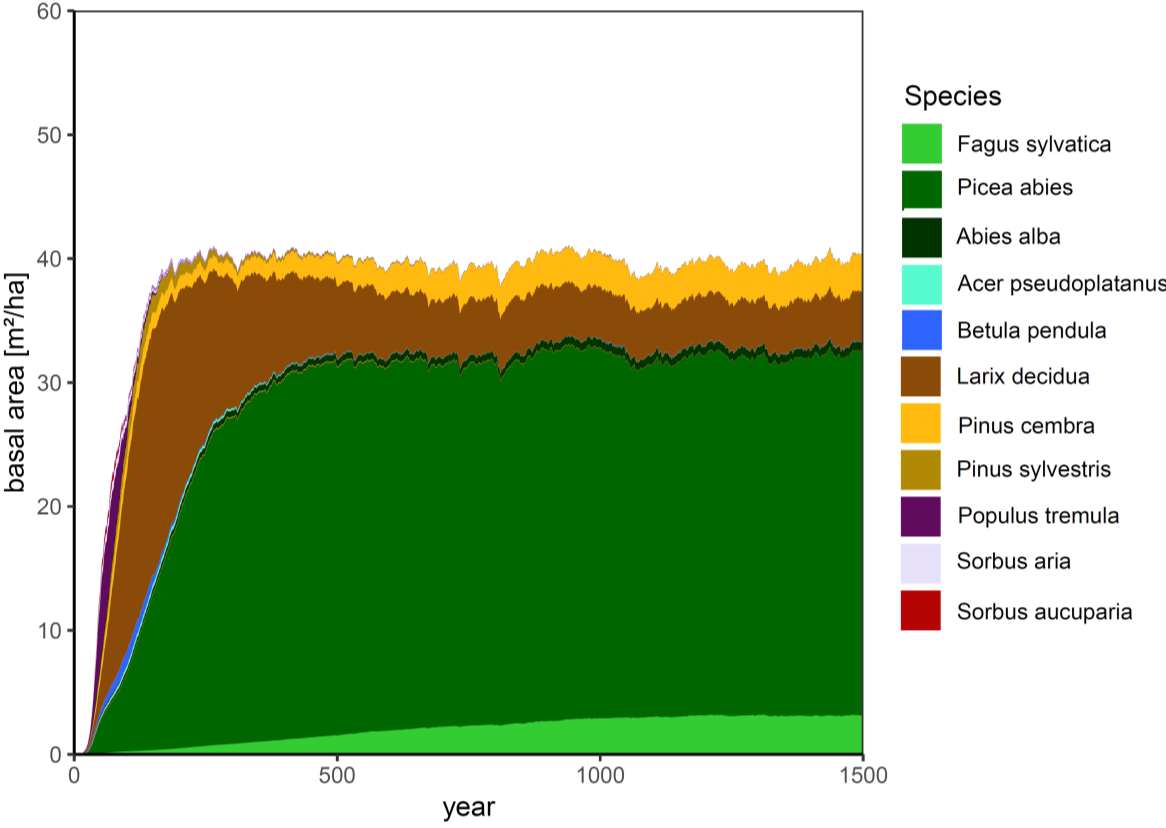
**

**Figure S1.4:** Development of succession over 1500 years in the absence of management and natural disturbances (Seidl et al. 2019).

**2. Intermediate topography scenario**

To further investigate the effect of topography, we designed a third, intermediate topography scenario. For this scenario, we rescaled our climate database so that all temperatures fell between the 25^th^ and 75^th^ percentile of the complex topography scenario (averaged over the entire historic climate data record from 1961-2014). The climate for each 1 ha pixel was mapped to the new range using quantile mapping. Soil variables were aggregated into larger groups to arrive at a smaller range and lower overall variability. We also tested the influence of the external seed area providing seed input in addition to mature trees already present on the landscape. This seed input serves as an important source of new species migrating into the landscape under a changing climate. In the case of the small seed area scenario, also used in the complex topography scenario (see main text), only the forest at the lowest elevation of the valley are receiving external seed input, with mountains and settled areas which surround the rest of the landscape blocking external seed input. The large seed area scenario represents equal seed input from all edges of the landscape. This seed area scenario was also used for the uniform topography scenario.
In the intermediate topography scenario, we simulated 10 replicates for each combination of precipitation change and seed area scenario, resulting in 80 simulation runs.

Overall, the climate response of the intermediate topography scenario lay between the complex and uniform scenarios, both for the individual indicator changes as well as with regard to the occurrence of tipping points (Fig. S1.5, Fig. S1.6). However, species dominance levels differ from the other scenarios at intermediate levels of landscape-scale climate and soil variation (Fig. S1.8). The seed area size mainly influences the variability between simulation replicates (higher with small seed area) and hysteresis.


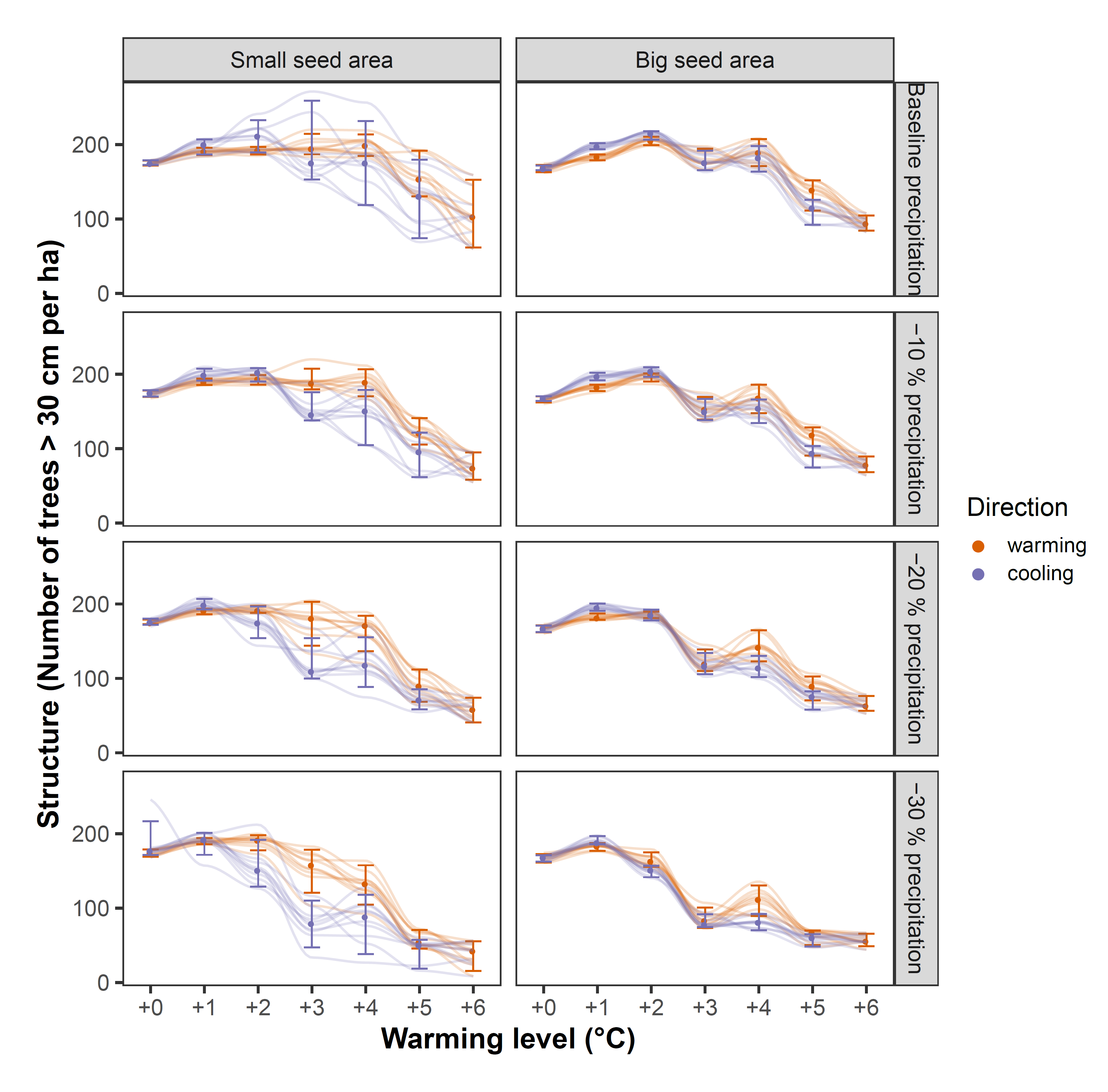


**Figure S1.5:** The response of forest structure (here described as the number of trees >30 cm in diameter) to climate warming (red) and subsequent cooling (purple) in the intermediate topography scenario. Values describe the state of the landscape after 1000 simulation years (median, 5th and 95th percentile across 10 replicates) and trajectories for all simulated replicates are shown. Trajectory lines are fitted using a LOESS model. Small seed area refers to external seed only entering from a small area at the bottom of the valley, large seed area means that the entire surrounding of the landscape acts as a seed source.


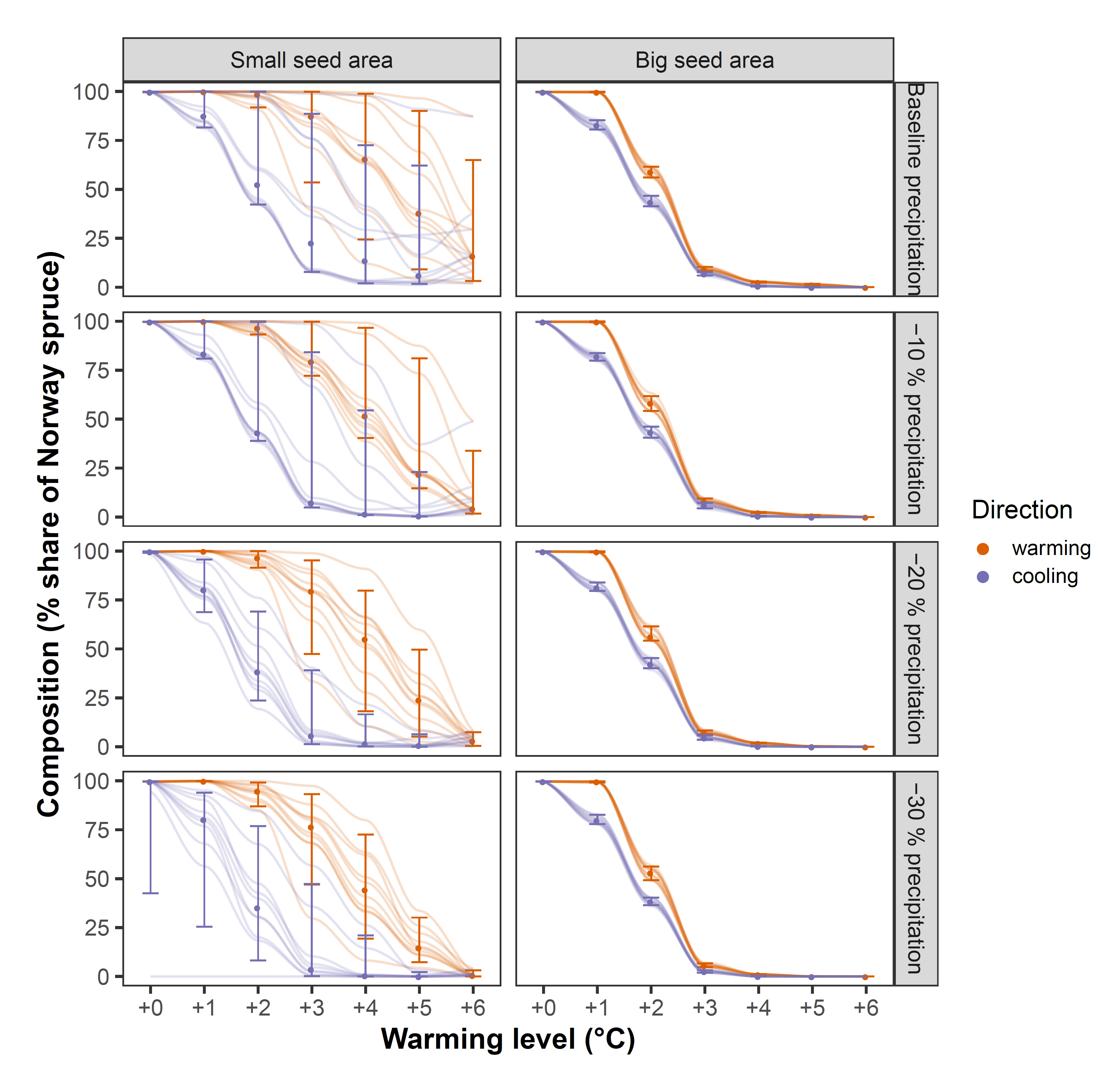


Figure S1.6: The response of forest composition (here described as the share of Norway spruce on total basal area) to climate warming (red) and subsequent cooling (purple) in the intermediate topography scenario. Values describe the state of the landscape after 1000 simulation years (median, 5th and 95th percentile across 10 replicates) and trajectories for all simulated replicates are shown. Trajectory lines are fitted using a LOESS model. Small seed area refers to external seed only entering from a small area at the bottom of the valley, large seed area means that the entire surrounding of the landscape acts as a seed source.


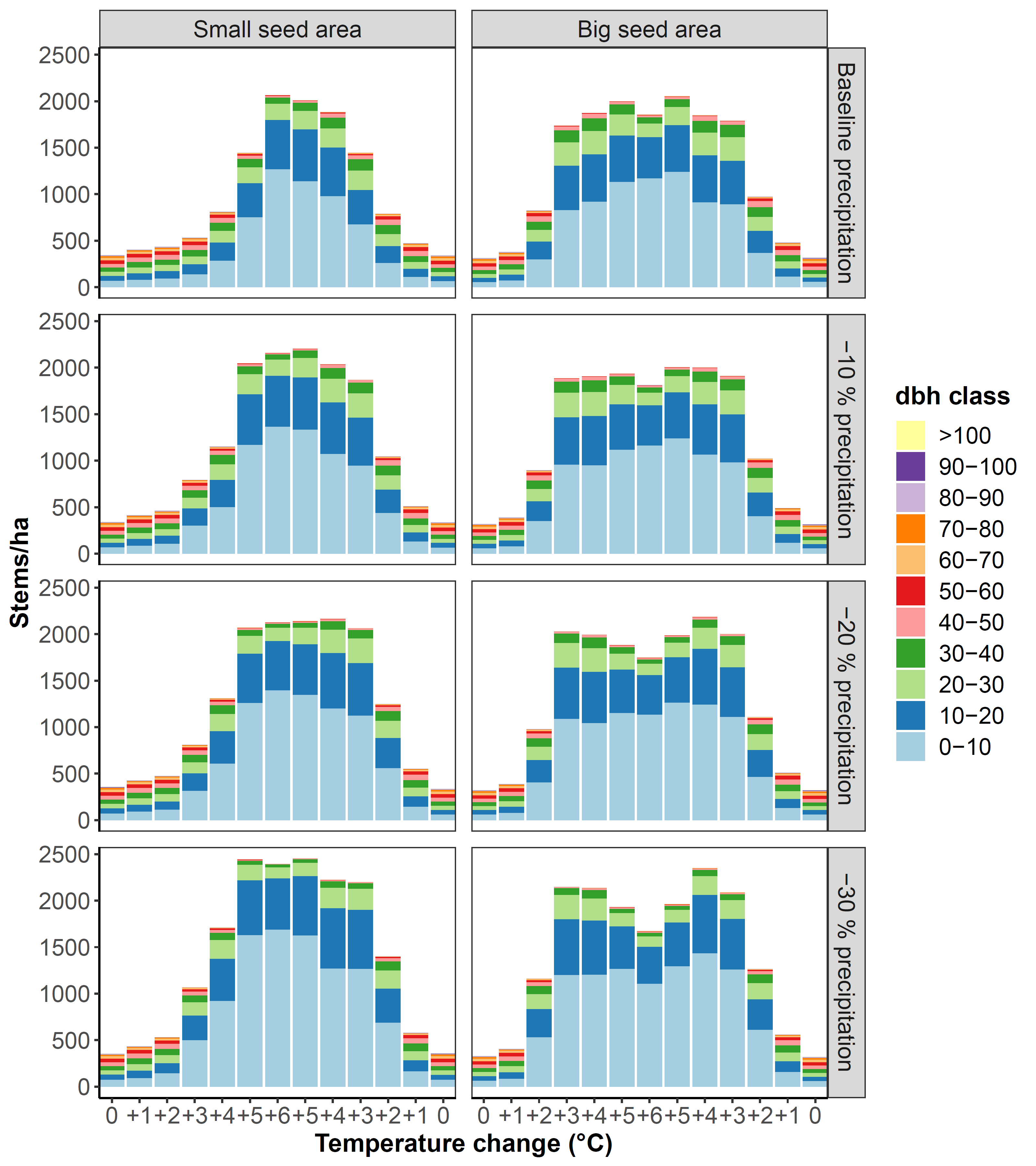


**Figure S1.7:** Simulated forest structure after 1000 simulation years at each temperature step (number of stems in DBH classes per ha) in the intermediate topography scenario. Small seed area refers to external seed only entering from a small area at the bottom of the valley, large seed area means that the entire surrounding of the landscape acts as a seed source.


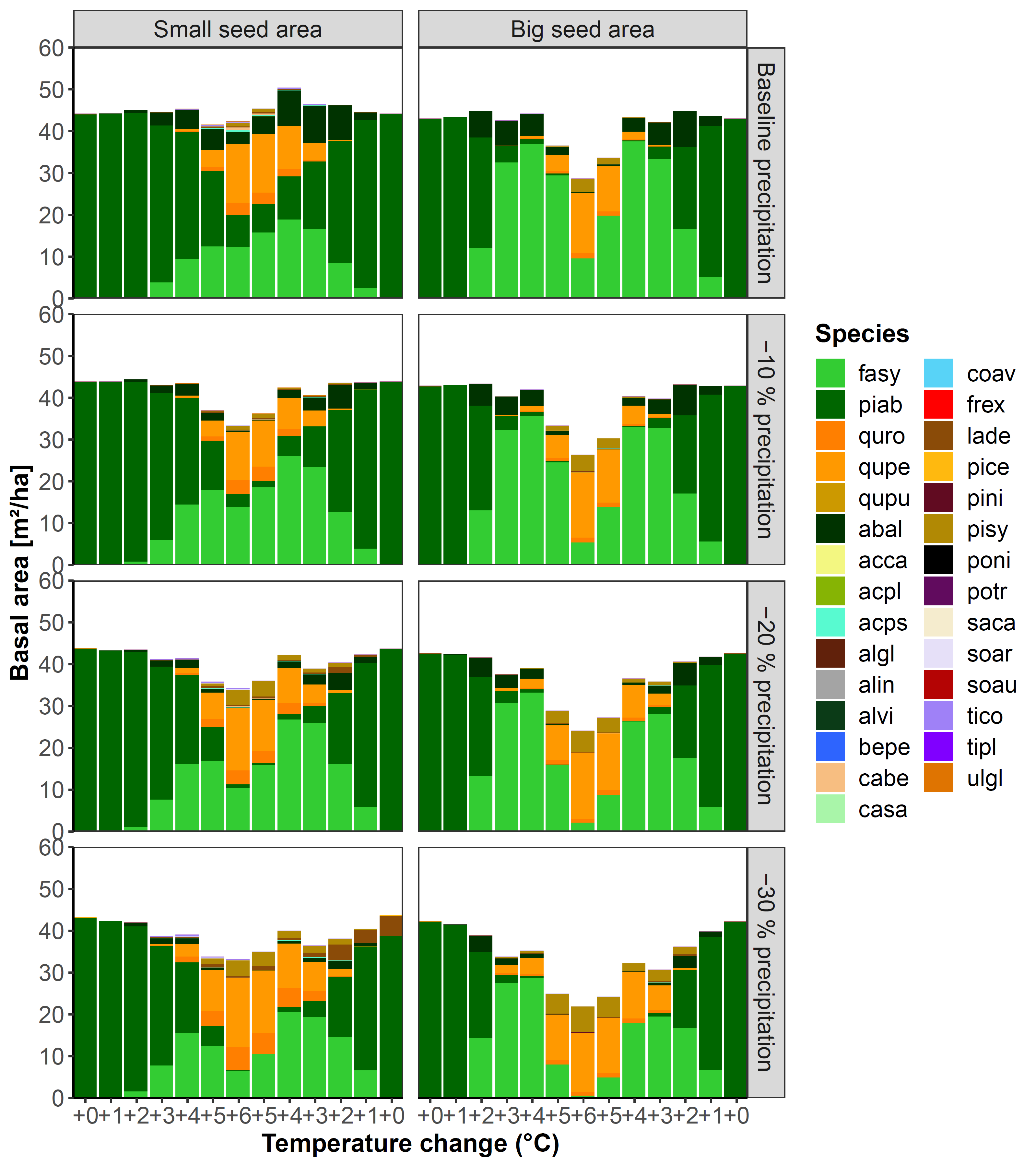


**Figure S1.8:** Simulated forest composition after 1000 simulation years at each temperature step in the intermediate topography scenario (basal area for each tree species). Species codes: abal=Abies alba, acca=Acer campestre, acpl=Acer platanoides, acps=Acer pseudoplatanus, algl=Alnus glutinosa, alin=Alnus incana, alvi=Alnus viridis, bepe=Betula pendula, cabe=Carpinus betulus, casa=Castanea sativa, coav=Corylus avellana, fasy=Fagus sylvatica, Frex=fraxinus excelsior, lade=Larix decidua, piab=Picea abies, pice=Pinus cembra, pini=Pinus nigra, pisy=Pinus sylvestris, poni=Populus nigra, potr=Populus tremula, qupe=Quercus petrea, qupu=Quercus pubescens, quro=Quercus robur, saca=Salix caprea, soar=Sorbus aria, soau=Sorbus aucuparia, tico=Tilia cordata, tipl=Tilia platyphyllos, ulgl=Ulmus glabra). Small seed area refers to external seed only entering from a small area at the bottom of the valley, large seed area means that the entire surrounding of the landscape acts as a seed source.


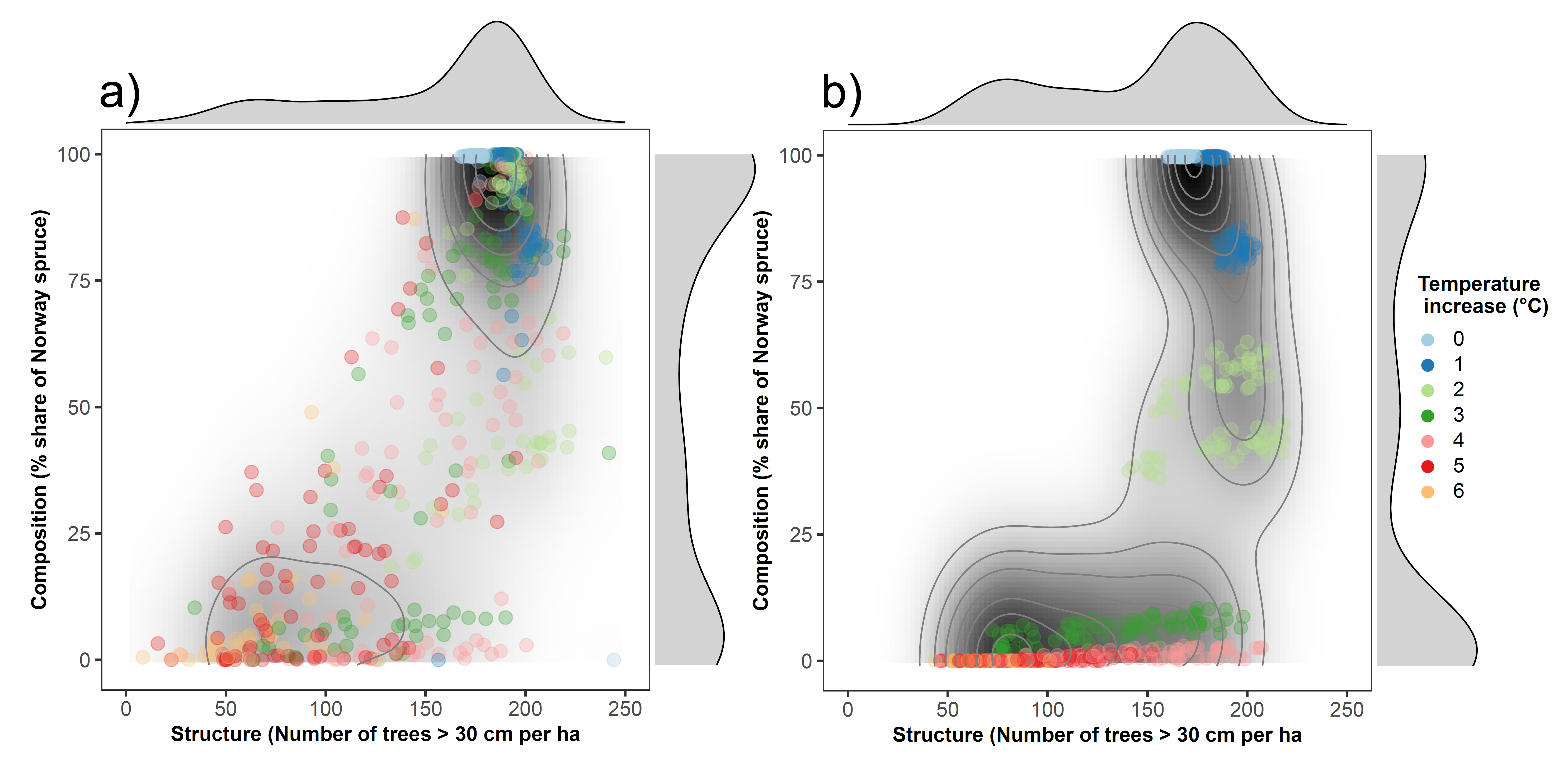


**Figure S1.9:** Location of the forest landscape in structure-composition attractor space for different warming levels and the small (a) and large (b) seed area scenarios over all precipitation scenarios in the intermediate topography scenario. Marginal plots and isolines indicate the probability density of all simulated cases.

**3. Sensitivity to different simulation designs**

3.1. Earlier reversal of climate forcing

**Figure S1.10:** The response of forest structure (here described as the number of trees >30 cm in diameter) to climate warming (red) and subsequent cooling (purple). Shown is one simulation trajectory at -20 % precipitation relative to historic climate. The temperature forcing is reversed at +4°C relative to historic climate here.

Figure S1.11: The response of forest composition (here described as the share of Norway spruce on total basal area) to climate warming (red) and subsequent cooling (purple). Shown is one simulation trajectory at -20 % precipitation relative to historic climate. The temperature forcing was reversed at +4°C relative to historic climate here.

**Figure S1.12:** Simulated forest structure after 1000 simulation years at each temperature step (number of stems in DBH classes per ha). Shown is one simulation at -20 % precipitation relative to historic climate. The temperature forcing was reversed at +4°C relative to historic climate here.

**Figure S1.13:** Simulated forest composition after 1000 simulation years at each temperature step (basal area for each tree species). Shown is one simulation at -20 % precipitation relative to historic climate. The temperature forcing was reversed at +4°C relative to historic climate here. Species codes: abal=Abies alba, acca=Acer campestre, acpl=Acer platanoides, acps=Acer pseudoplatanus, algl=Alnus glutinosa, alin=Alnus incana, alvi=Alnus viridis, bepe=Betula pendula, cabe=Carpinus betulus, casa=Castanea sativa, coav=Corylus avellana, fasy=Fagus sylvatica, Frex=fraxinus excelsior, lade=Larix decidua, piab=Picea abies, pice=Pinus cembra, pini=Pinus nigra, pisy=Pinus sylvestris, poni=Populus nigra, potr=Populus tremula, qupe=Quercus petrea, qupu=Quercus pubescens, quro=Quercus robur, saca=Salix caprea, soar=Sorbus aria, soau=Sorbus aucuparia, tico=Tilia cordata, tipl=Tilia platyphyllos, ulgl=Ulmus glabra)

3.2. Longer simulation durations at stable temperature

**Figure S1.14:** Stem diameter distribution after 1000 simulation years (averaged over the simulation years 950-1000) and 2000 simulation years (averaged over the simulation years 1950-2000) at +0°C relative to historic climate. Shown are single runs at baseline precipitation and -20% precipitation relative to historic climate. Simulations were run for a total of 2000 years under historic mean temperature.

**Figure S1.15:** Comparison of species composition after 1000 simulation years (averaged over the simulation years 950-1000) and 2000 simulation years (averaged over the simulation years 1950-2000) at +0°C relative to historic climate. Shown are single runs at baseline precipitation and -20% precipitation relative to historic climate. Simulations were run for a total of 2000 years under historic mean temperature.

**Figure S1.16:** Stem diameter distribution after 1000 simulation years (averaged over the period from 950-1000) and 2000 simulation years (averaged over the period from 1950-2000) at +3°C relative to historic climate. Shown are single runs at baseline precipitation and -20% precipitation relative to historic climate. Simulations were run for a total of 2000 years at +3°C relative to historic climate.

**Figure S1.17:** Comparison of species composition after 1000 simulation years (averaged over the simulation years 950-1000) and 2000 simulation years (averaged over the simulation years 1950-2000) at +3°C relative to historic climate. Shown are single runs at baseline precipitation and -20% precipitation relative to historic climate. Simulations were run for a total of 2000 years at +3°C relative to historic climate.


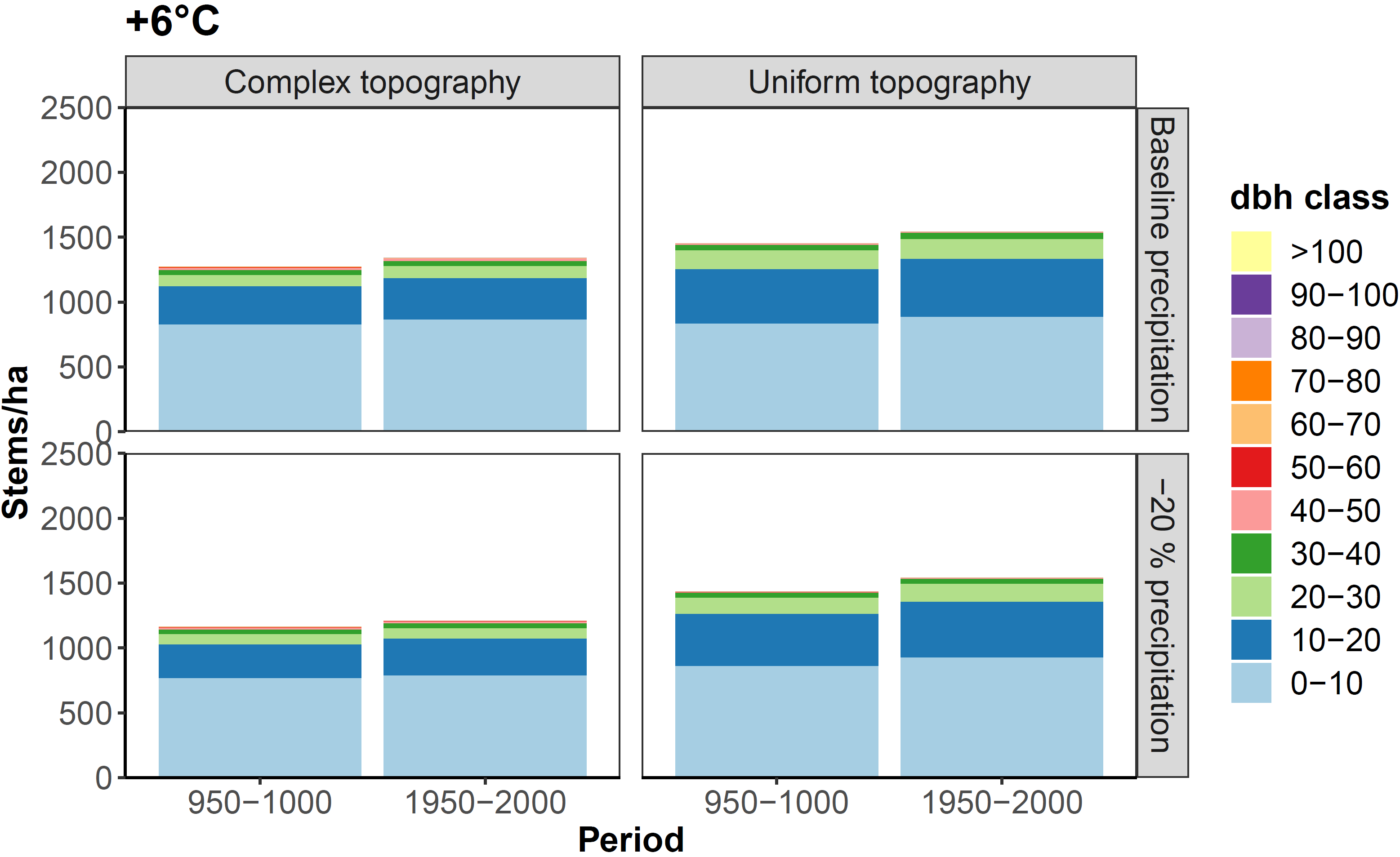


**Figure S1.18:** Stem diameter distribution after 1000 simulation years (averaged over the simulation years 950-1000) and 2000 simulation years (averaged over the simulation years 1950-2000) at +6°C relative to historic climate. Shown are single runs at baseline precipitation and -20% precipitation relative to historic climate. Simulations were run for a total of 2000 years at +3°C relative to historic climate.

**Figure S1.19:** Comparison of species composition after 1000 simulation years (averaged over the simulation years 950-1000) and 2000 simulation years (averaged over the simulation years 1950-2000) at +6°C relative to historic climate. Shown are single runs at baseline precipitation and -20% precipitation relative to historic climate. Simulations were run for a total of 2000 years at +6°C relative to historic climate.

3.3. Bigger increments of warming within 1000-year time steps

**Figure S1.20:** The response of forest structure (here described as the number of trees >30 cm in diameter) to climate warming (red) and subsequent cooling (purple). Shown is one simulation at -20 % precipitation relative to historic climate. Temperature was raised by two degrees each 1000 years. The landscape failed to equilibrate to a 2°C change within 1000 years, we therefore did not draw the connecting lines.

**Figure S1.21:** The response of forest composition (here described as the share of Norway spruce on total basal area) to climate warming (red) and subsequent cooling (purple). Shown is one simulation at -20 % precipitation relative to historic climate. Temperature was raised by two degrees each 1000 years. The landscape failed to equilibrate to a 2°C change within 1000 years, we therefore did now draw the connecting lines.

**Figure S1.22:** Simulated forest structure after 1000 simulation years at each temperature step (number of stems in DBH classes per ha). Shown is one simulation at -20 % precipitation relative to historic climate. Temperature was raised by two degrees each 1000 years. The landscape failed to equilibrate to a 2°C change within 1000 years.

**Figure S1.23:** Simulated forest composition after 1000 simulation years at each temperature step (basal area for each tree species). Shown is one simulation at -20 % precipitation relative to historic climate. Temperature was raised by two degrees each 1000 years. The landscape failed to equilibrate to a 2°C change within 1000 years. Species codes: abal=Abies alba, acca=Acer campestre, acpl=Acer platanoides, acps=Acer pseudoplatanus, algl=Alnus glutinosa, alin=Alnus incana, alvi=Alnus viridis, bepe=Betula pendula, cabe=Carpinus betulus, casa=Castanea sativa, coav=Corylus avellana, fasy=Fagus sylvatica, Frex=fraxinus excelsior, lade=Larix decidua, piab=Picea abies, pice=Pinus cembra, pini=Pinus nigra, pisy=Pinus sylvestris, poni=Populus nigra, potr=Populus tremula, qupe=Quercus petrea, qupu=Quercus pubescens, quro=Quercus robur, saca=Salix caprea, soar=Sorbus aria, soau=Sorbus aucuparia, tico=Tilia cordata, tipl=Tilia platyphyllos, ulgl=Ulmus glabra)

**4. Simulation design**

Figure S1.24: Sequence of temperature change (mean change across sampled period) during the simulation.

**5. Sensitivity analysis for alternative indicator definitions**

5.1. Forest structure: Varying diameter thresholds


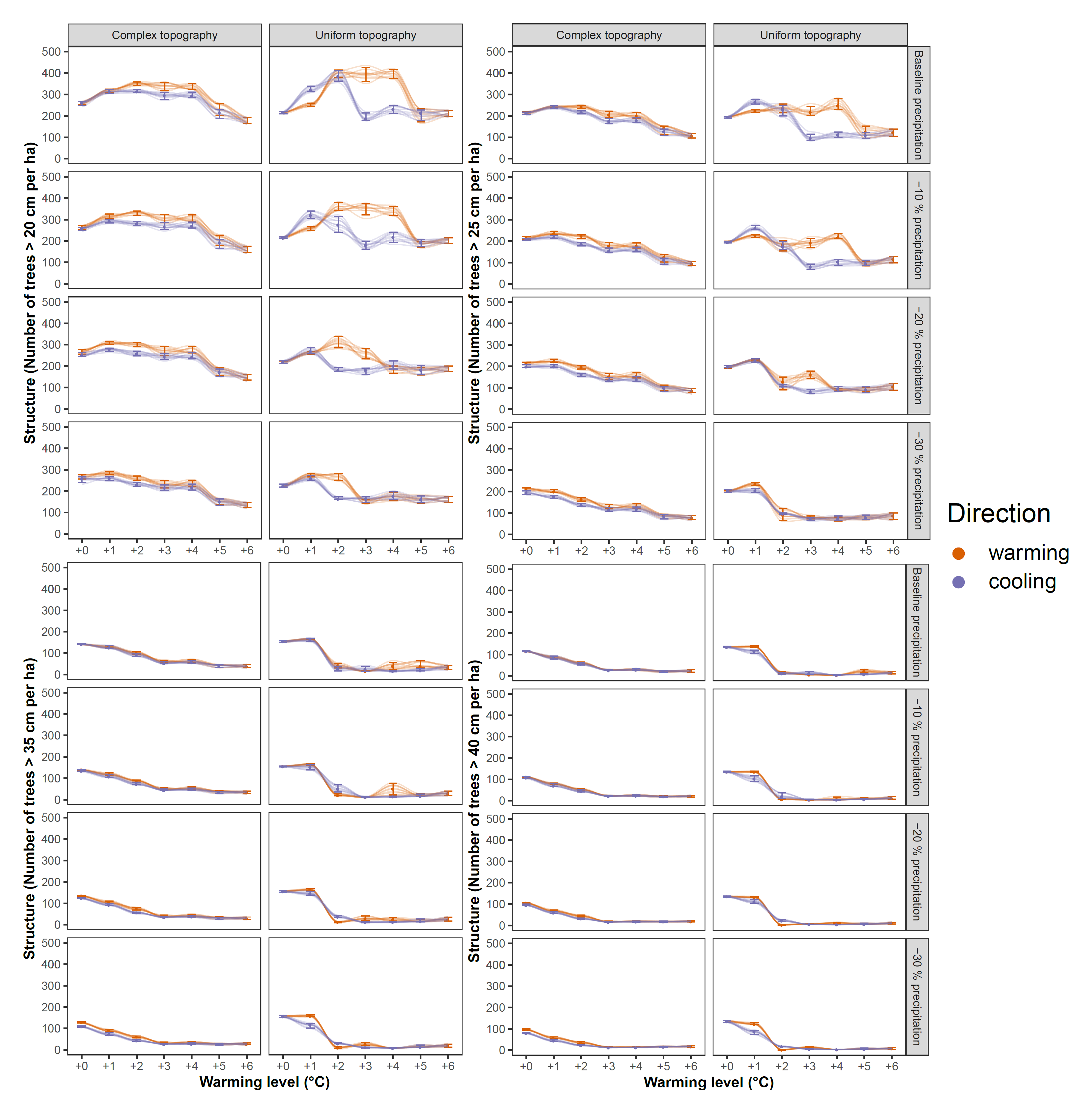


Figure S1.25: Sensitivity to different diameter thresholds used in the definition of the forest structure indicator. Shown are thresholds of 20 cm (top left panels), 25 cm (top right), 35 cm (bottom left) and 40 cm (bottom right). Lines give the number of trees per hectare larger than the respective threshold value.

S5.2. Forest composition: Alternative definition of the indicator


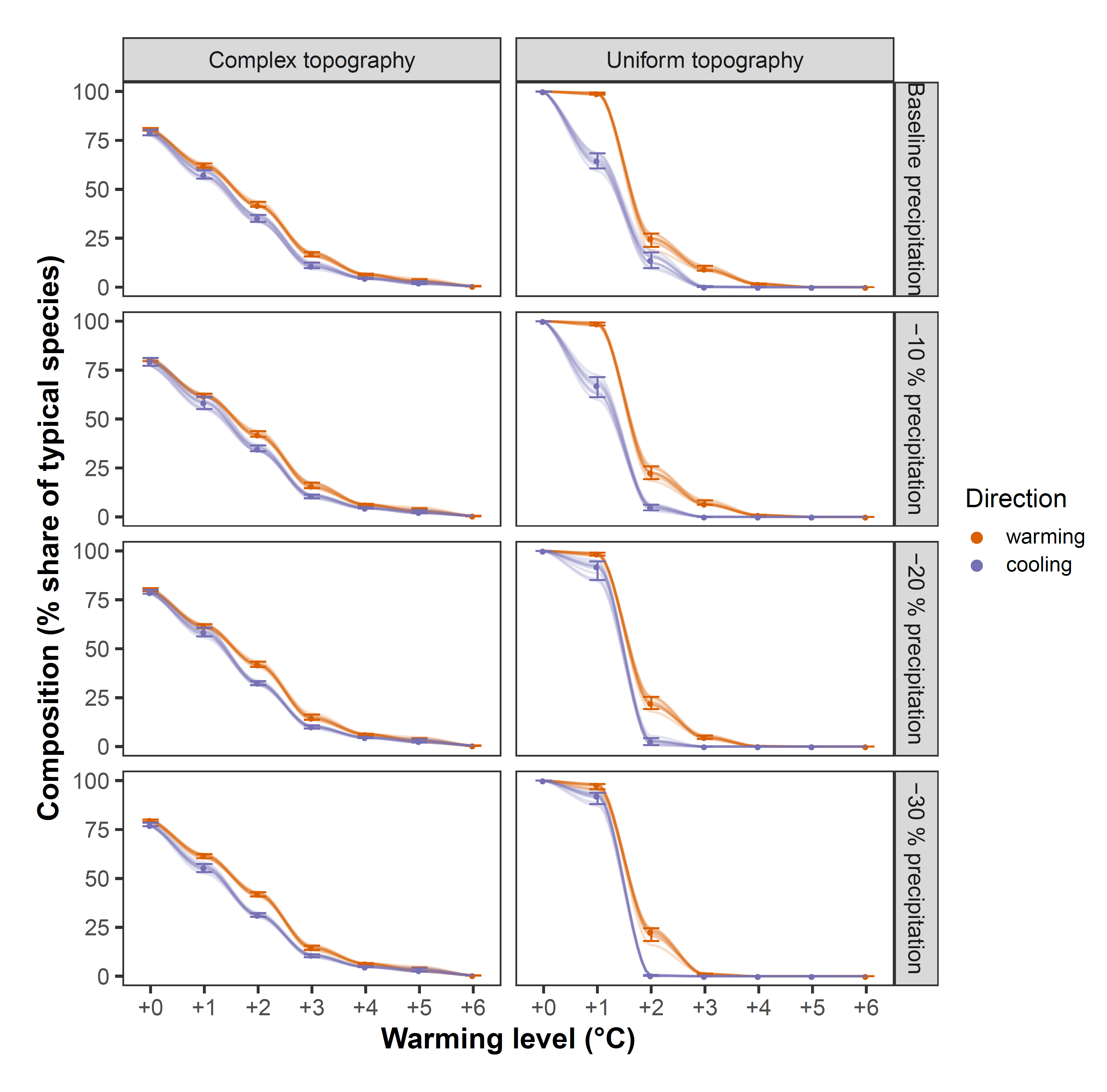


Figure S1.26: Response to warming when including additional species (Larix decidua and Pinus cembra) in the indicator used to define forest composition.

**6. Changes in forest structure and composition**


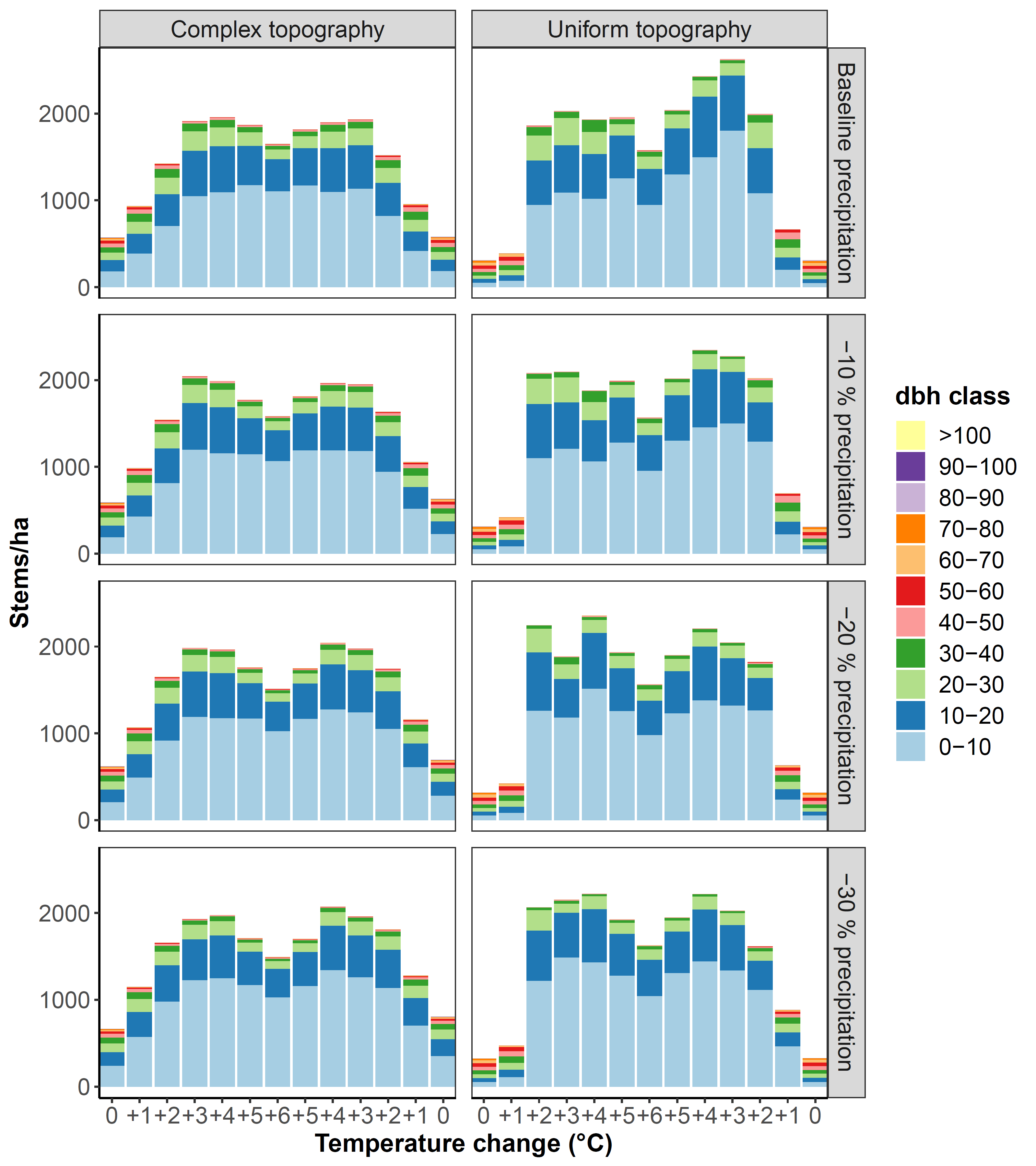


Figure S1.27: Simulated forest structure after 1000 simulation years at each temperature step (number of stems in DBH classes per ha).


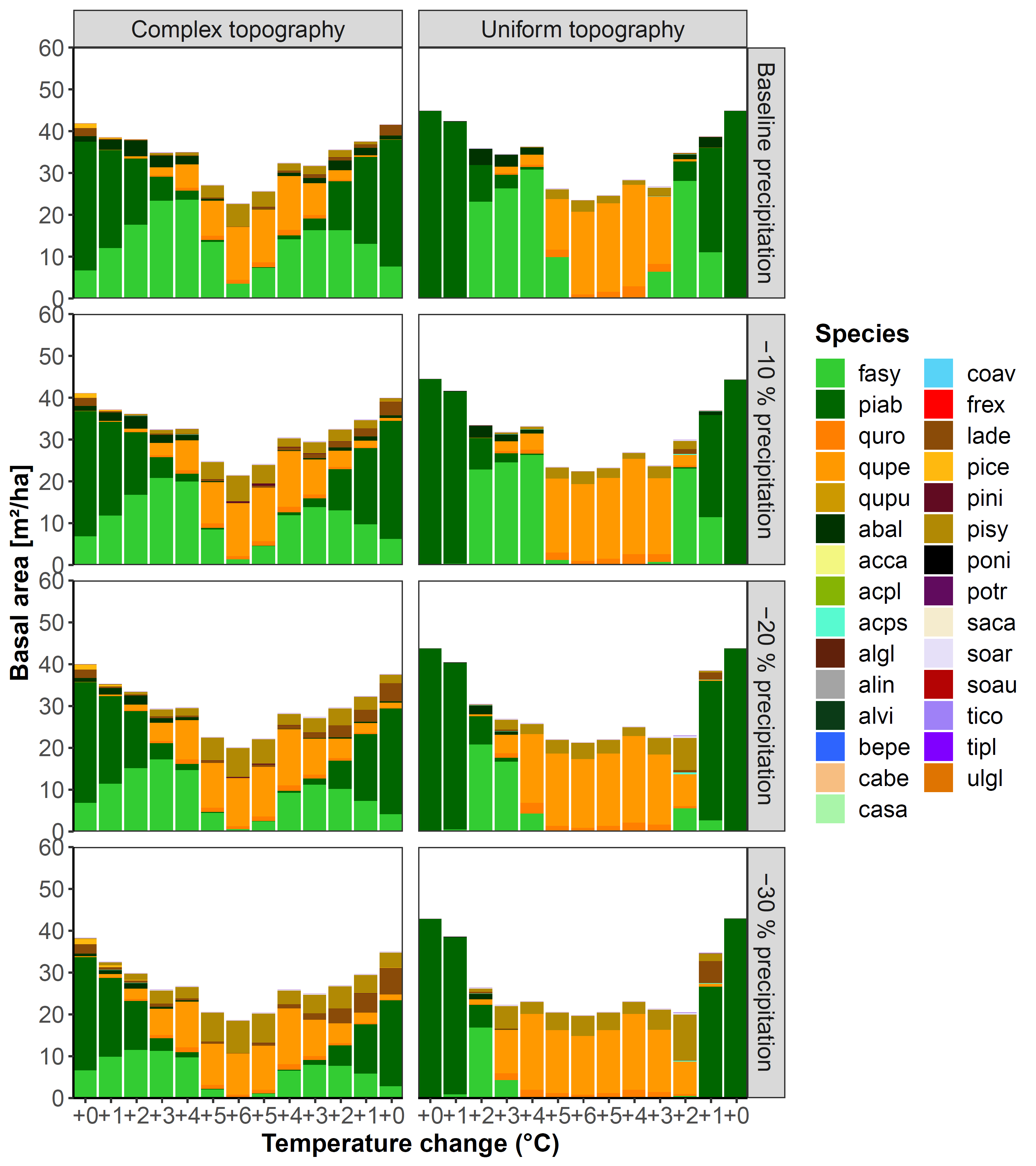
**Figure S1.28:** Simulated forest composition after 1000 simulation years at each temperature step (basal area for each tree species). Species codes: abal=Abies alba, acca=Acer campestre, acpl=Acer platanoides, acps=Acer pseudoplatanus, algl=Alnus glutinosa, alin=Alnus incana, alvi=Alnus viridis, bepe=Betula pendula, cabe=Carpinus betulus, casa=Castanea sativa, coav=Corylus avellana, fasy=Fagus sylvatica, Frex=fraxinus excelsior, lade=Larix decidua, piab=Picea abies, pice=Pinus cembra, pini=Pinus nigra, pisy=Pinus sylvestris, poni=Populus nigra, potr=Populus tremula, qupe=Quercus petrea, qupu=Quercus pubescens, quro=Quercus robur, saca=Salix caprea, soar=Sorbus aria, soau=Sorbus aucuparia, tico=Tilia cordata, tipl=Tilia platyphyllos, ulgl=Ulmus glabra)

**7. Development of forest composition and structure across elevational bands**


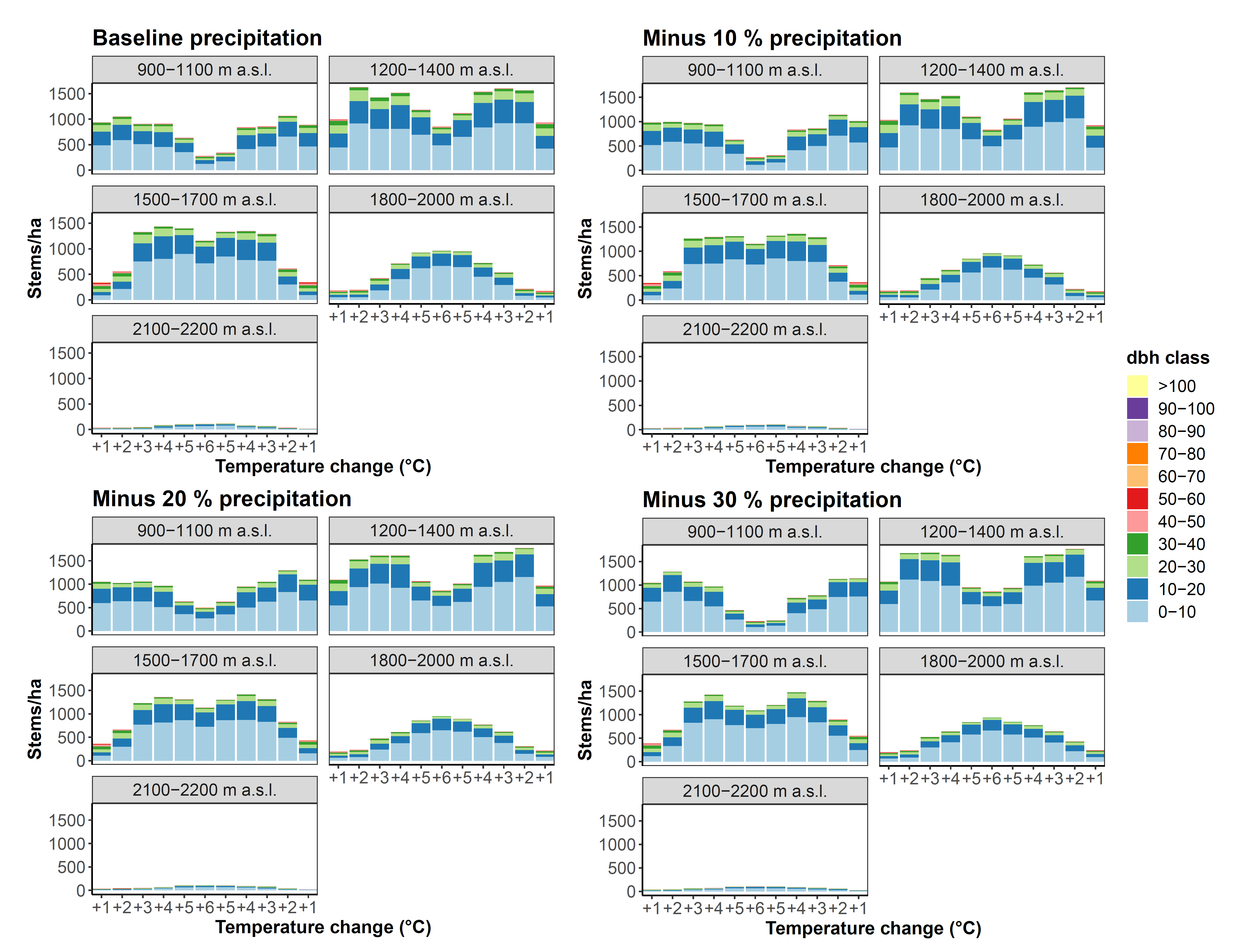


**Figure S1.29:** Simulated forest structure after 1000 simulation years at each temperature step (number of stems in DBH classes per ha across 300m elevational bands. Shown is one simulation per precipitation scenario.


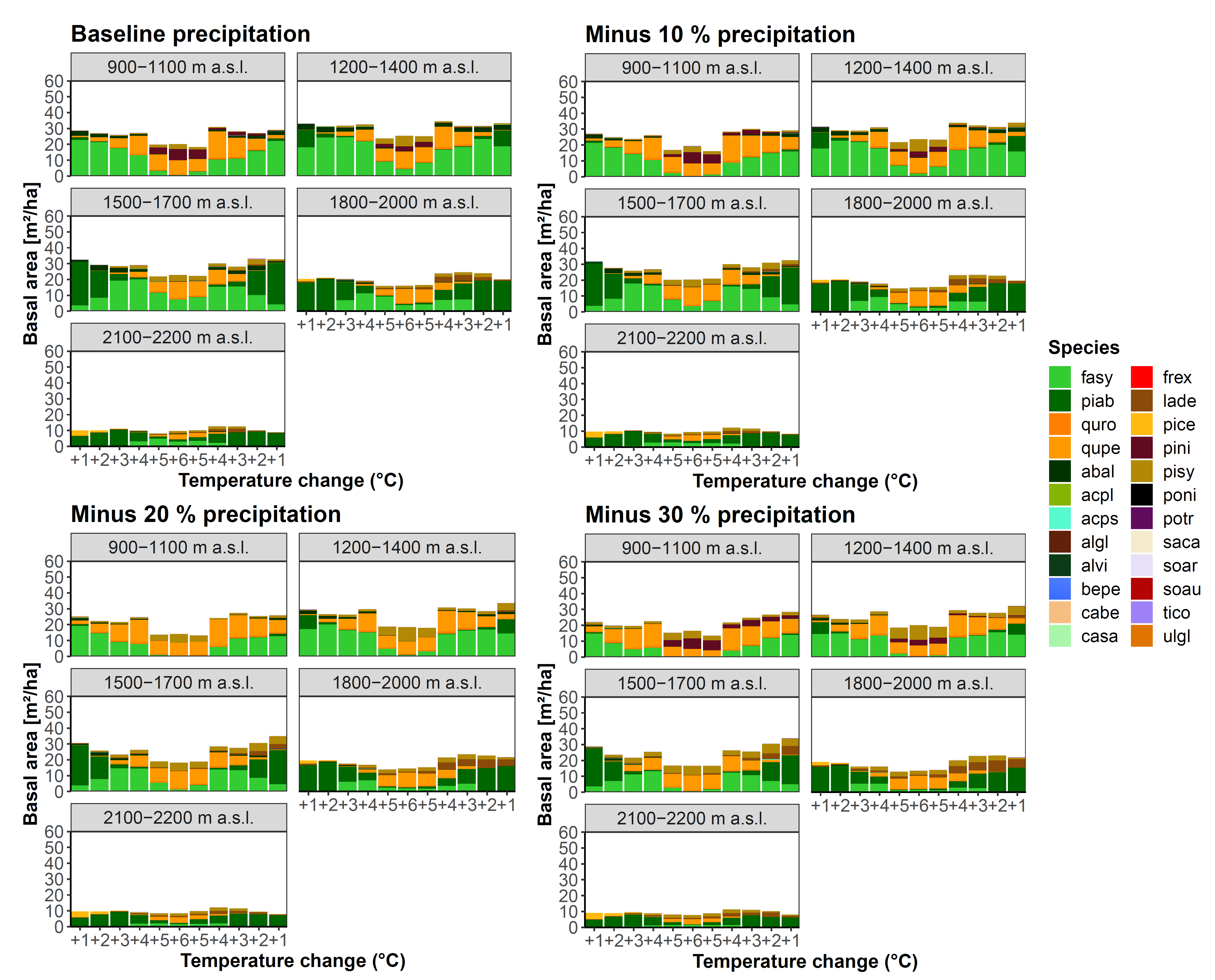


**Figure S1.30:** Simulated forest composition after 1000 simulation years at each temperature step (basal area for each tree species) across 300m elevational bands. Shown is one simulation per precipitation scenario. Species codes: abal=Abies alba, acca=Acer campestre, acpl=Acer platanoides, acps=Acer pseudoplatanus, algl=Alnus glutinosa, alin=Alnus incana, alvi=Alnus viridis, bepe=Betula pendula, cabe=Carpinus betulus, casa=Castanea sativa, coav=Corylus avellana, fasy=Fagus sylvatica, Frex=fraxinus excelsior, lade=Larix decidua, piab=Picea abies, pice=Pinus cembra, pini=Pinus nigra, pisy=Pinus sylvestris, poni=Populus nigra, potr=Populus tremula, qupe=Quercus petrea, qupu=Quercus pubescens, quro=Quercus robur, saca=Salix caprea, soar=Sorbus aria, soau=Sorbus aucuparia, tico=Tilia cordata, tipl=Tilia platyphyllos, ulgl=Ulmus glabra)

8: Potential drivers of hysteresis

Table S1.1: Optimal and minimal temperature for tree growth by tree species as well as the difference between these temperature points illustrating the temperature amplitude for tree growth of each species.

| **Species** | **Optimal temperature (°C)** | **Minimal temperature (°C)** | **Amplitude (°C)** |
| --- | --- | --- | --- |
| Abies alba | 21 | 0 | 21 |
| Acer campestre | 24 | 3 | 21 |
| Acer platanoides | 24 | 3 | 21 |
| Acer pseudoplatanus | 21 | 3 | 18 |
| Alnus glutinosa | 20 | 2 | 18 |
| Alnus incana | 22 | 3 | 19 |
| Alnus viridis | 18 | 1 | 17 |
| Betula pendula | 17 | 0 | 17 |
| Carpinus betulus | 23 | 5 | 18 |
| Castanea sativa | 25 | 5 | 20 |
| Corylus avellana | 22 | 3 | 19 |
| Fagus sylvatica | 19 | 3 | 16 |
| Fraxinus excelsior | 20 | 3 | 17 |
| Larix decidua | 19 | -1 | 20 |
| Picea abies | 17 | -2 | 19 |
| Pinus cembra | 11 | 1 | 10 |
| Pinus nigra | 25 | 1 | 24 |
| Pinus sylvestris | 23 | 1 | 22 |
| Populus nigra | 21 | 2 | 19 |
| Populus tremula | 21 | 2 | 19 |
| Quercus petraea | 23 | 5 | 18 |
| Quercus pubescence | 23 | 5 | 18 |
| Quercus robur | 23 | 5 | 18 |
| Salix caprea | 21 | 1 | 20 |
| Sorbus aria | 22 | 3 | 19 |
| Sorbus aucuparia | 22 | 3 | 19 |
| Tilia cordata | 24 | 5 | 19 |
| Tilia platyphyllos | 24 | 5 | 19 |
| Ulmus glabra | 24 | 3 | 21 |
