# Supplementary material for: Climate change causes critical transitions and irreversible alterations of mountain forests
Source: Glob Chang Biol. 2020 May 8;26(7):4013–27. doi: 10.1111/gcb.15118 (PMC7317840; doi:10.1111/gcb.15118)
Supplement: Supplementary file 2 — supinfo S2 [file GCB-26-4013-s002.pdf]

Supplementary Material S2 for:

Climate change causes critical transitions and irreversible alterations of mountain forests

Katharina Albrich, Werner Rammer, Rupert Seidl

Spatial development of indicators

Pages 2-9: Share of Norway Spruce

Pages 10-17: Number of Stems larger than 30 cm per ha

All values averaged across 10 replicates for each 1 ha (100x100 m) pixel over 50 years at the end of each 1000 years simulation at each temperature step.

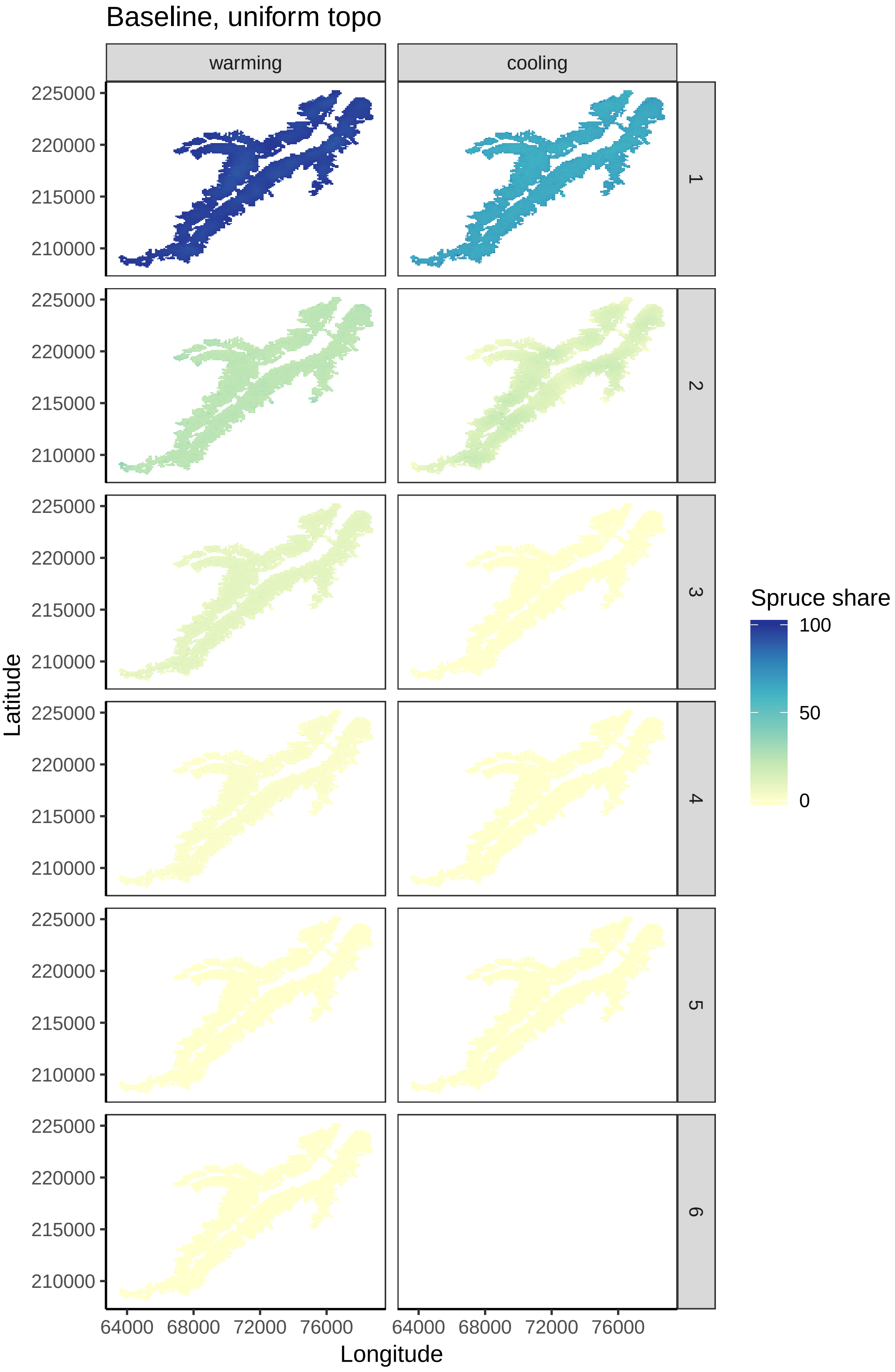

Baseline, uniform topo
warming
cooling
1
2
3
4
5
6
225000
220000
215000
210000
Latitude
64000
68000
72000
76000
Longitude
Spruce share
100
50
0

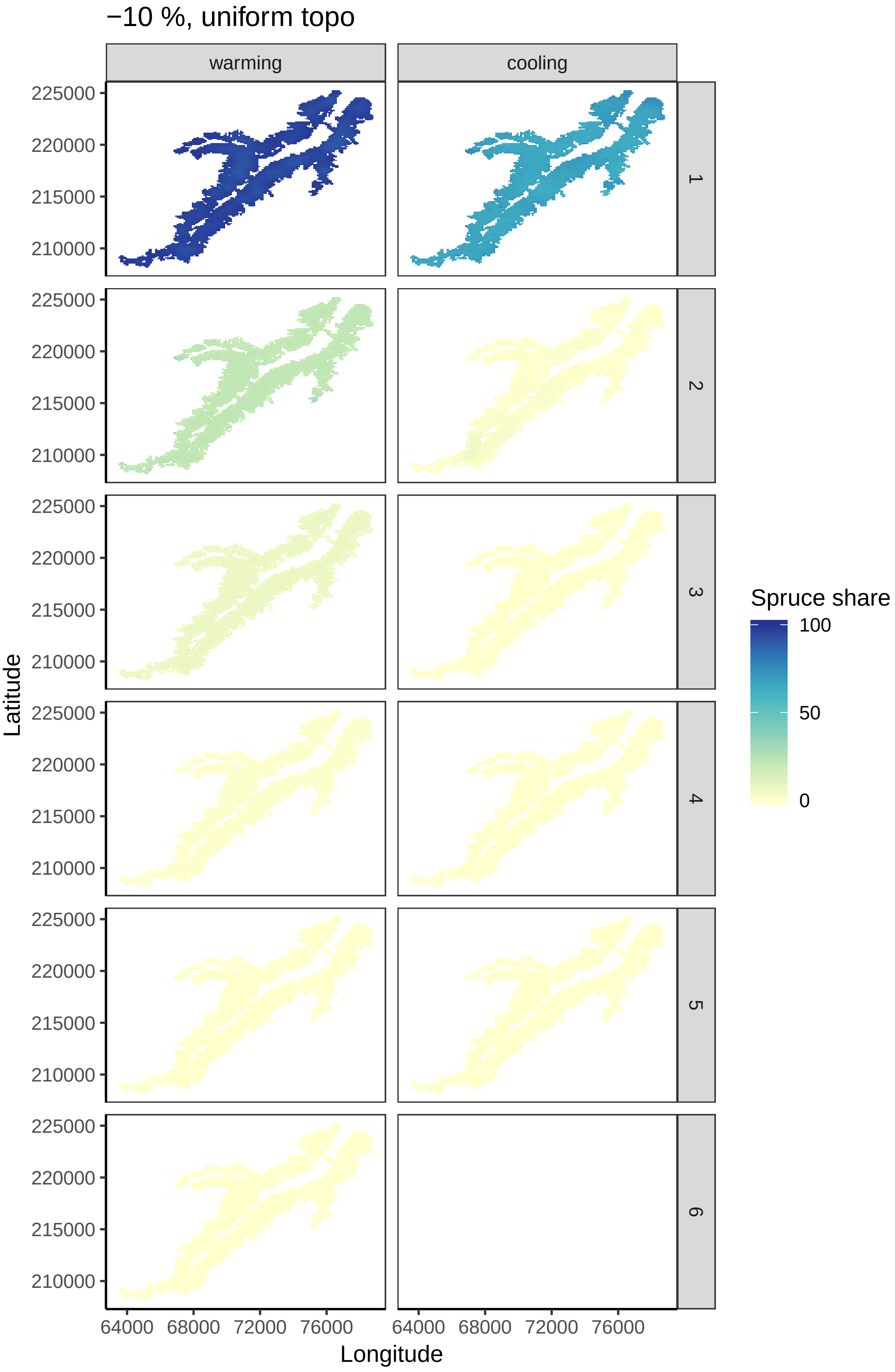
−10 %, uniform topo
warming
cooling
1
2
3
4
5
6
225000
220000
215000
210000
64000
68000
72000
76000
Longitude
Latitude
Spruce share
100
50
0

−20 %, uniform topo

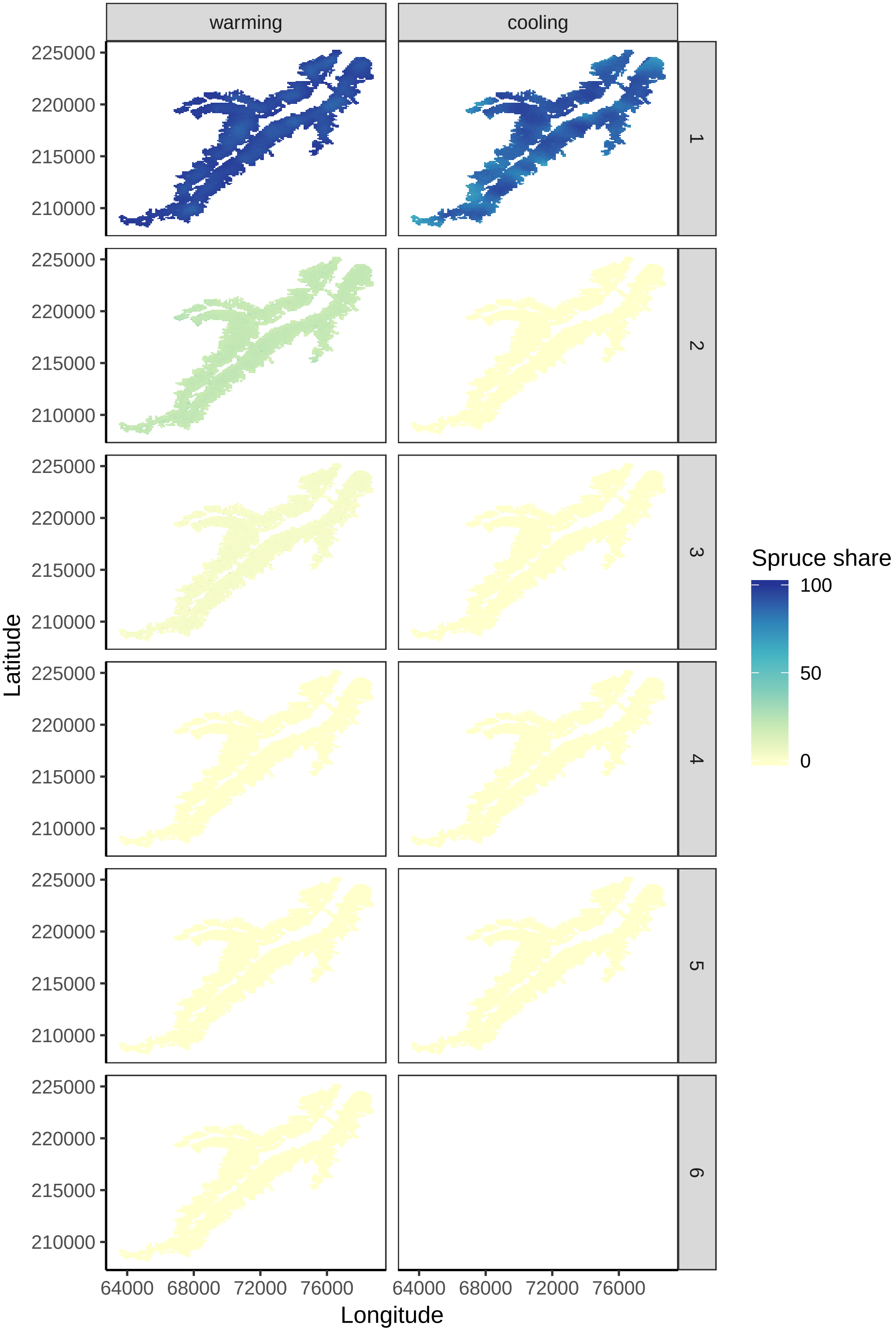

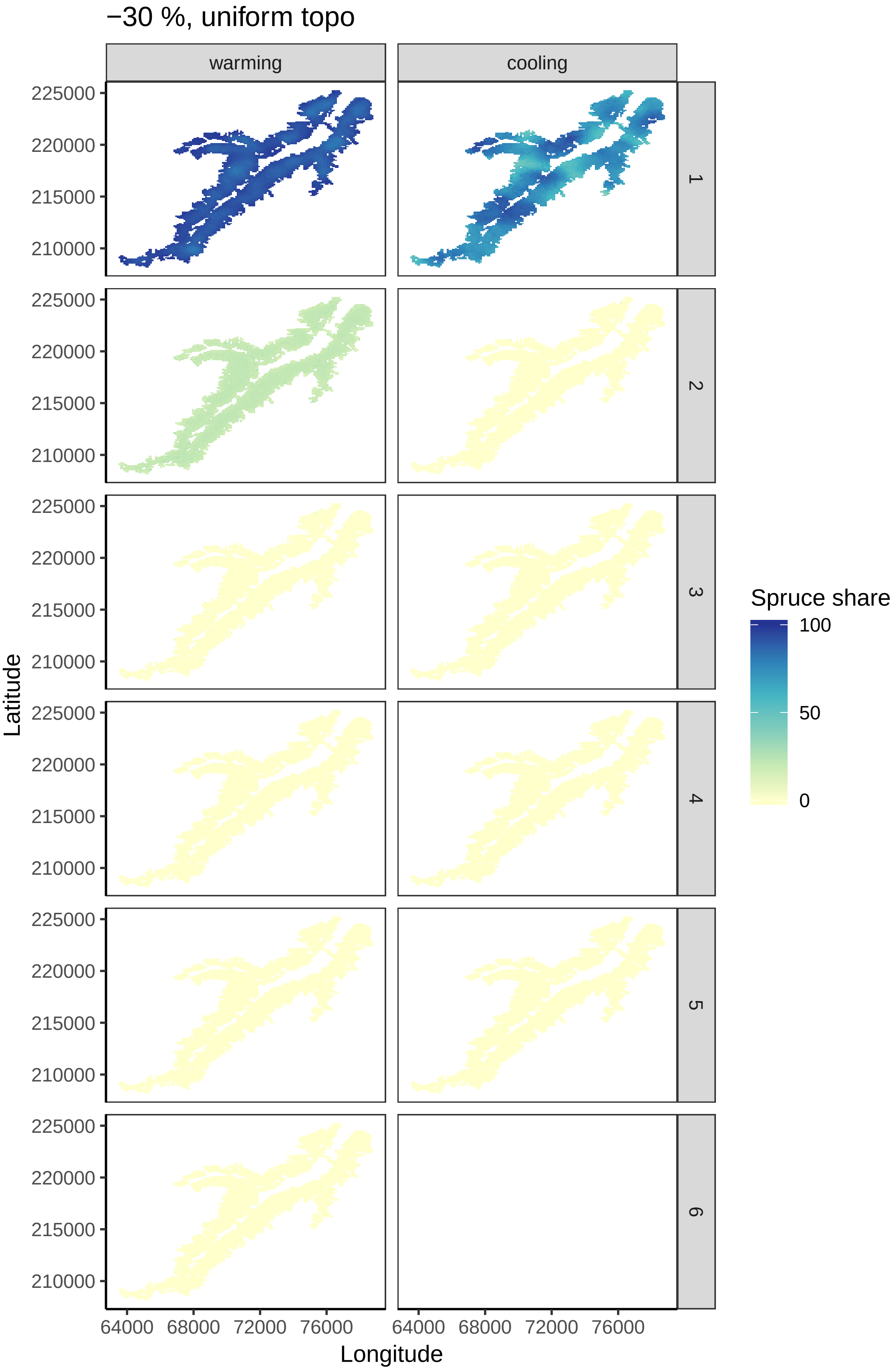

−30 %, uniform topo
warming
cooling
1
2
3
4
5
6
225000
220000
215000
210000
Latitude
64000
68000
72000
76000
Longitude
Spruce share
100
50
0

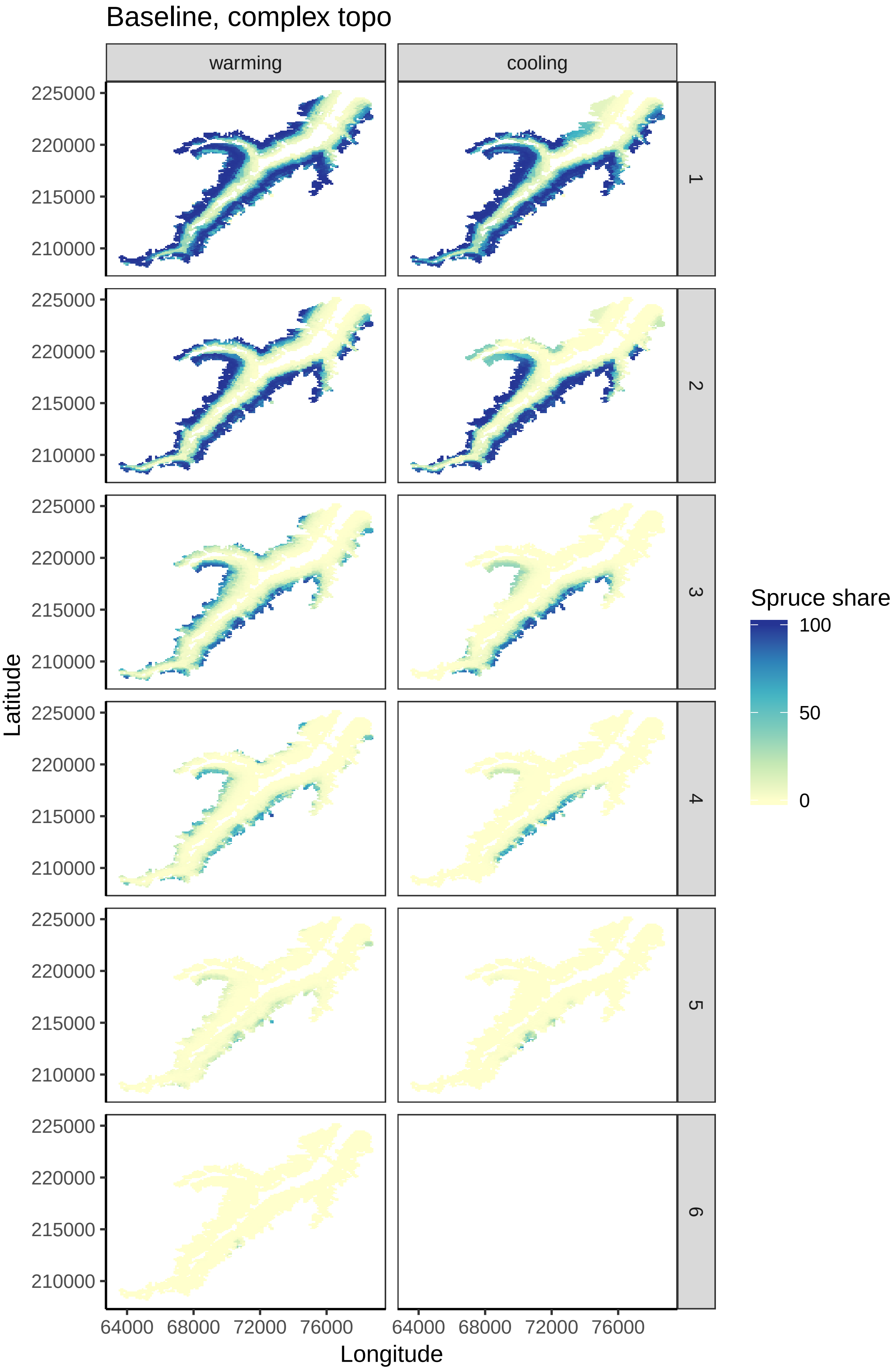

Baseline, complex topo
warming
cooling
1
2
3
4
5
6
225000
220000
215000
210000
Latitude
64000
68000
72000
76000
Longitude
Spruce share
100
50
0

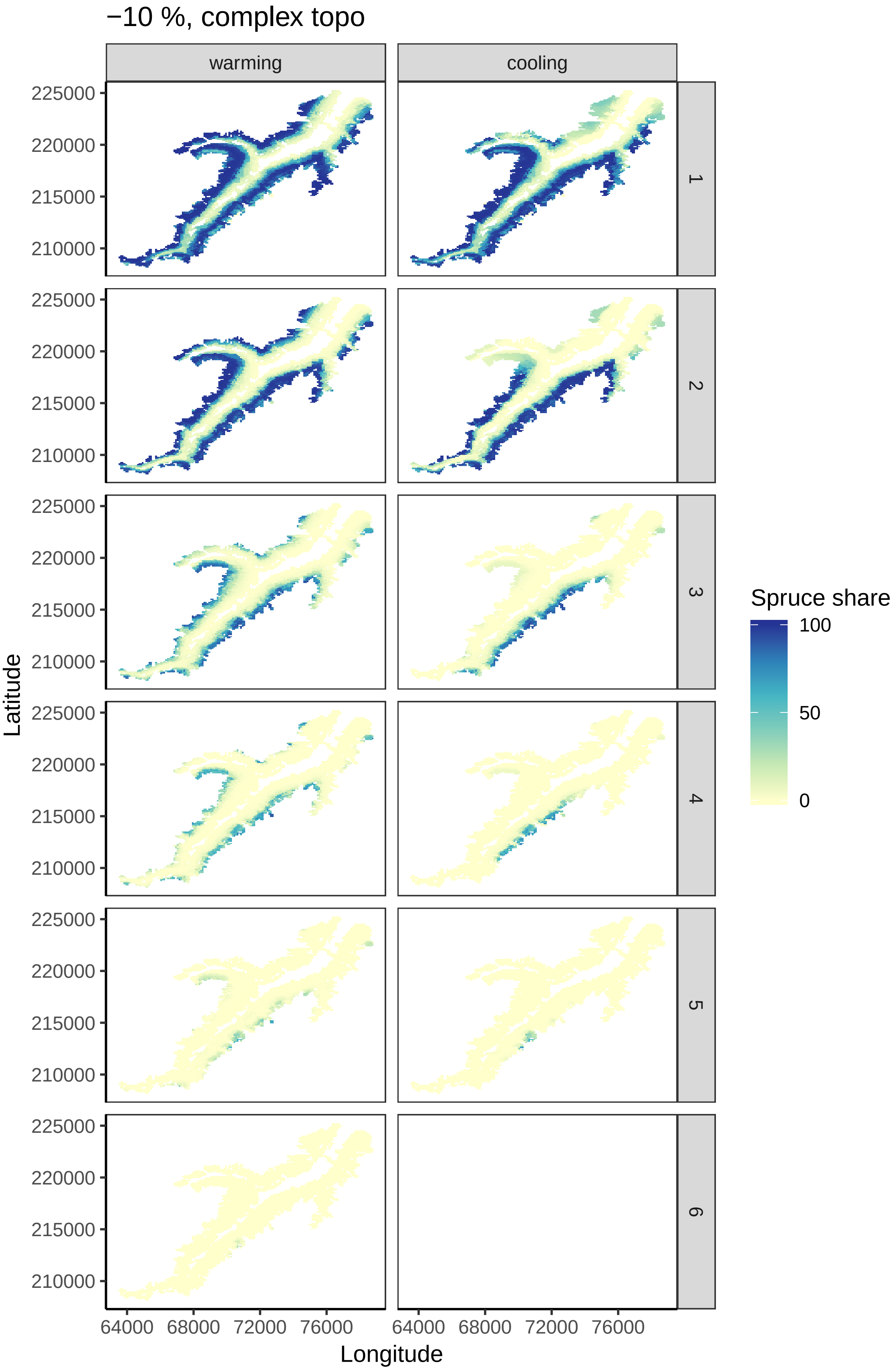

−10 %, complex topo
warming
cooling
1
2
3
4
5
6
225000
220000
215000
210000
Latitude
64000
68000
72000
76000
Longitude
Spruce share
100
50
0

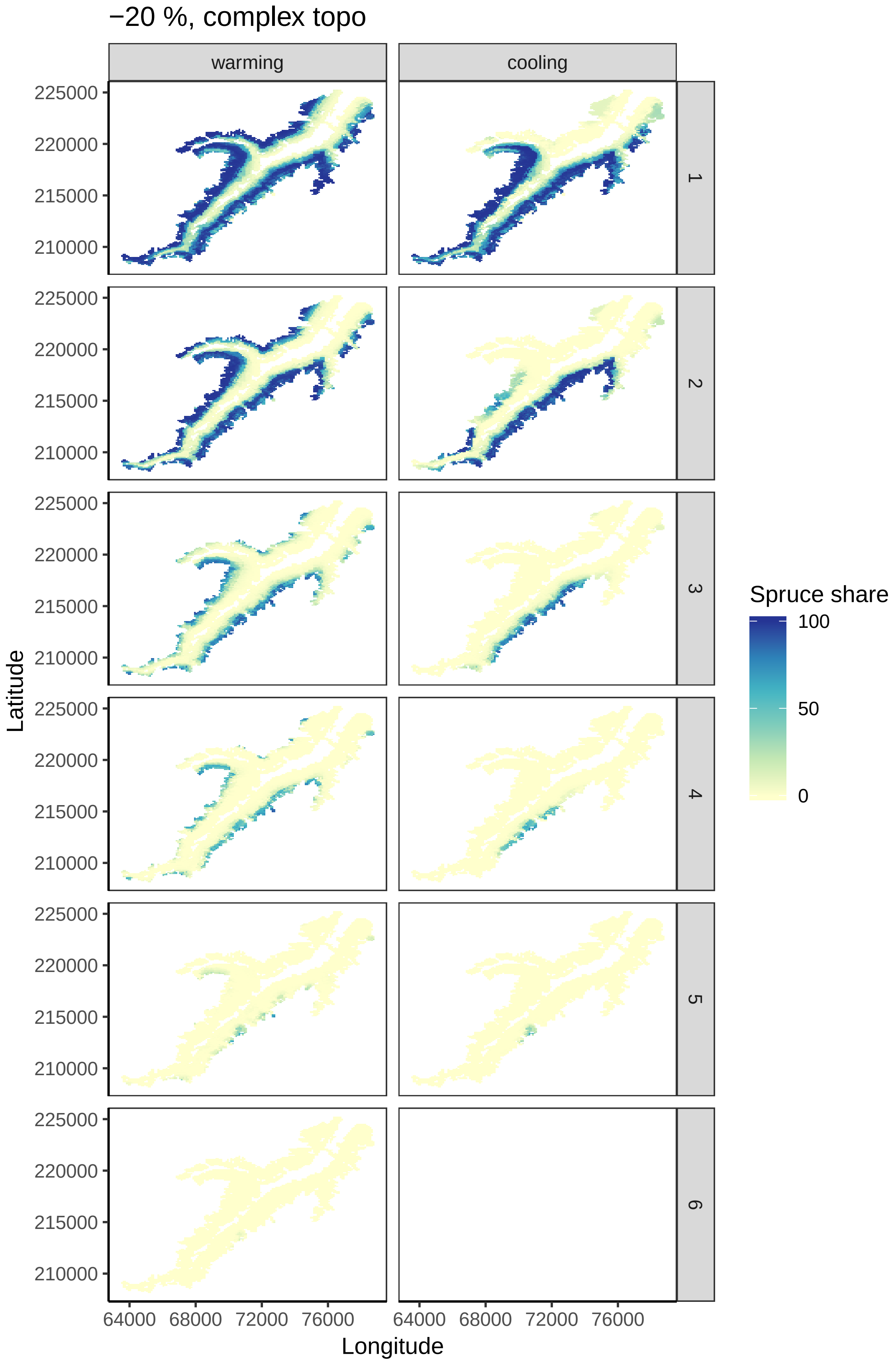

−20 %, complex topo
warming
cooling
1
2
3
4
5
6
225000
220000
215000
210000
Latitude
64000
68000
72000
76000
Longitude
Spruce share
100
50
0

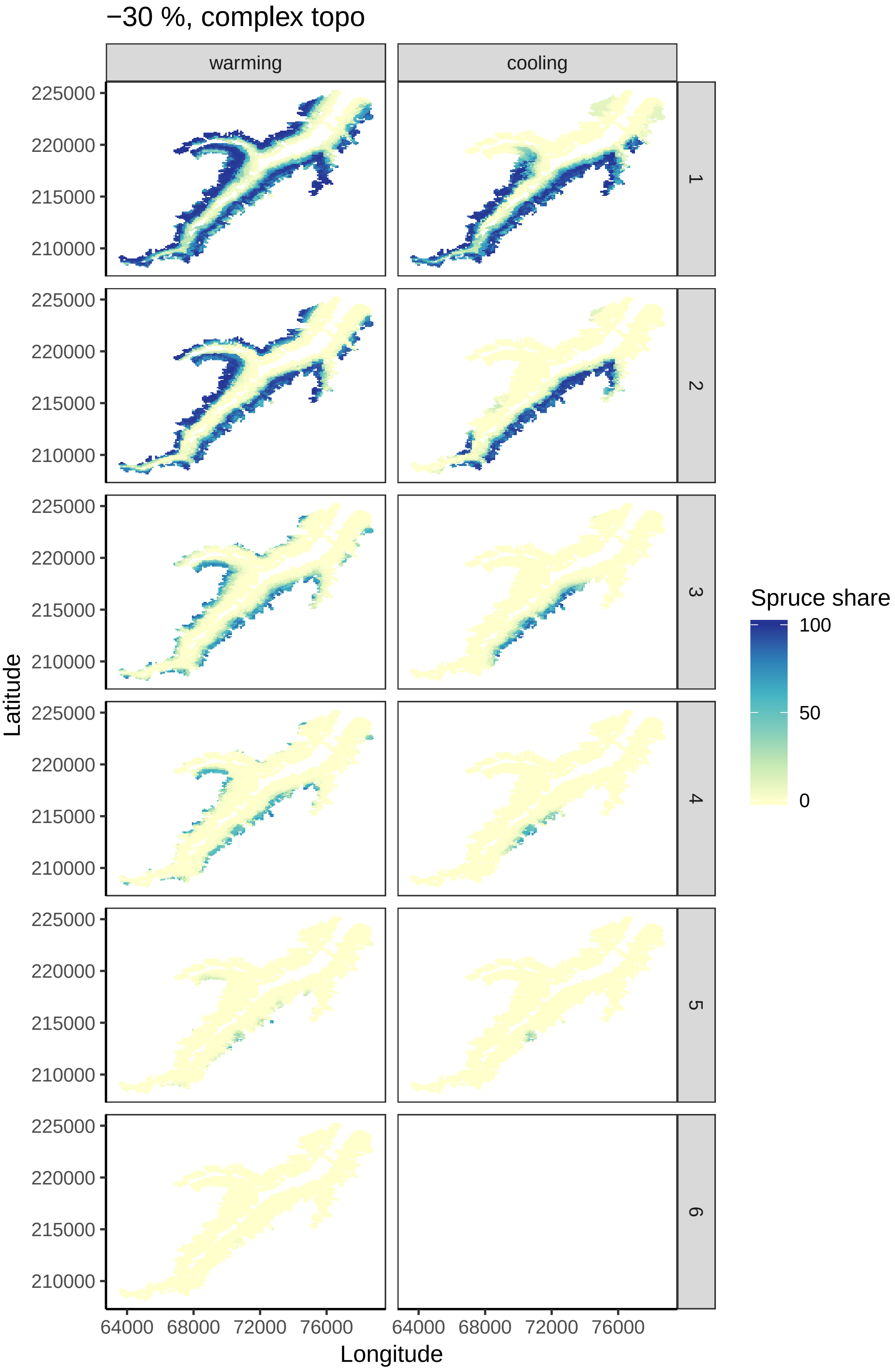

−30 %, complex topo
warming
cooling
1
2
3
4
5
6
225000
220000
215000
210000
Latitude
64000
68000
72000
76000
Longitude
Spruce share
100
50
0

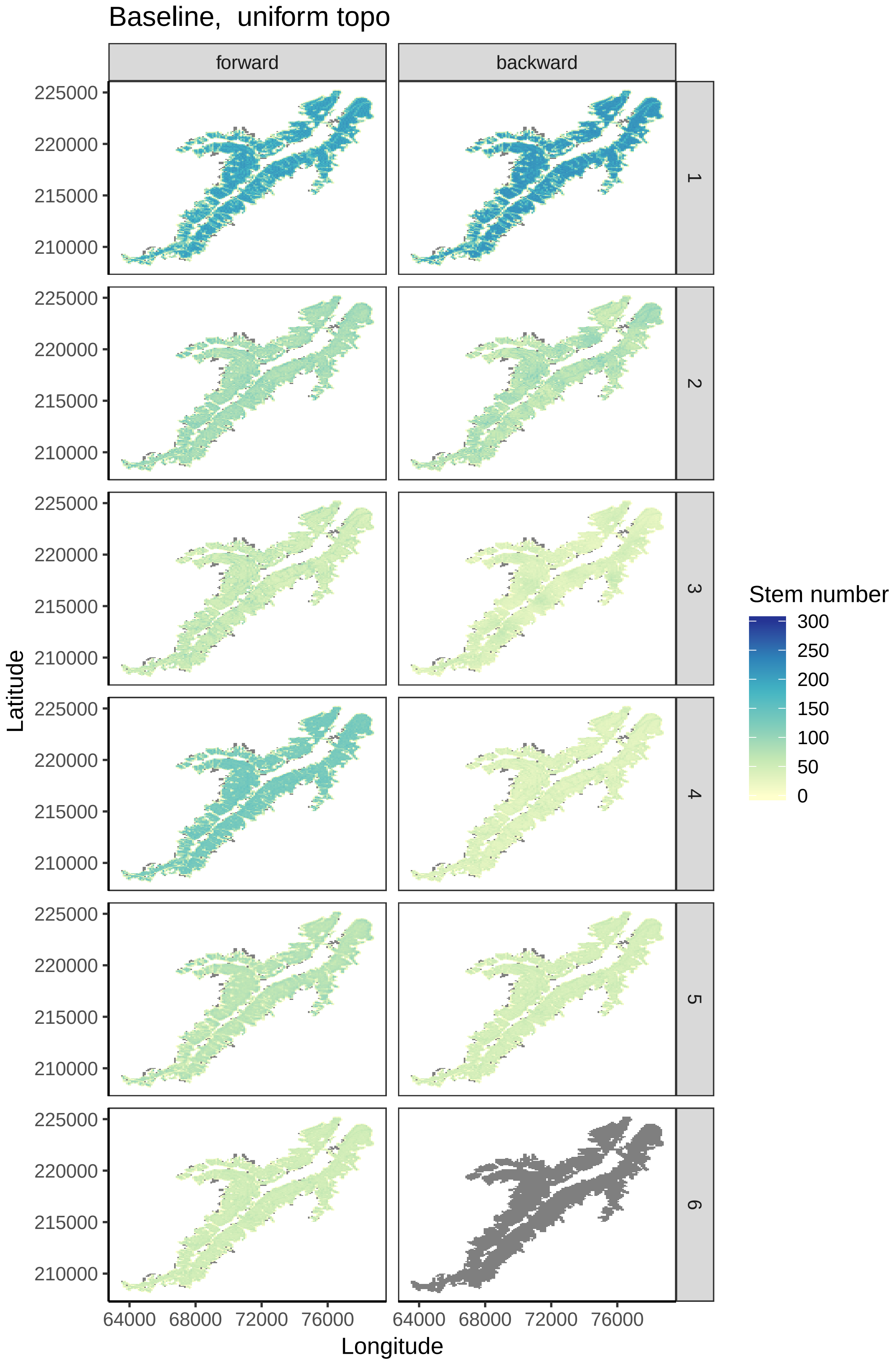

Baseline, uniform topo
forward
backward
1
2
3
4
5
6
Latitude
225000
220000
215000
210000
Longitude
64000
68000
72000
76000
Stem number
300
250
200
150
100
50
0

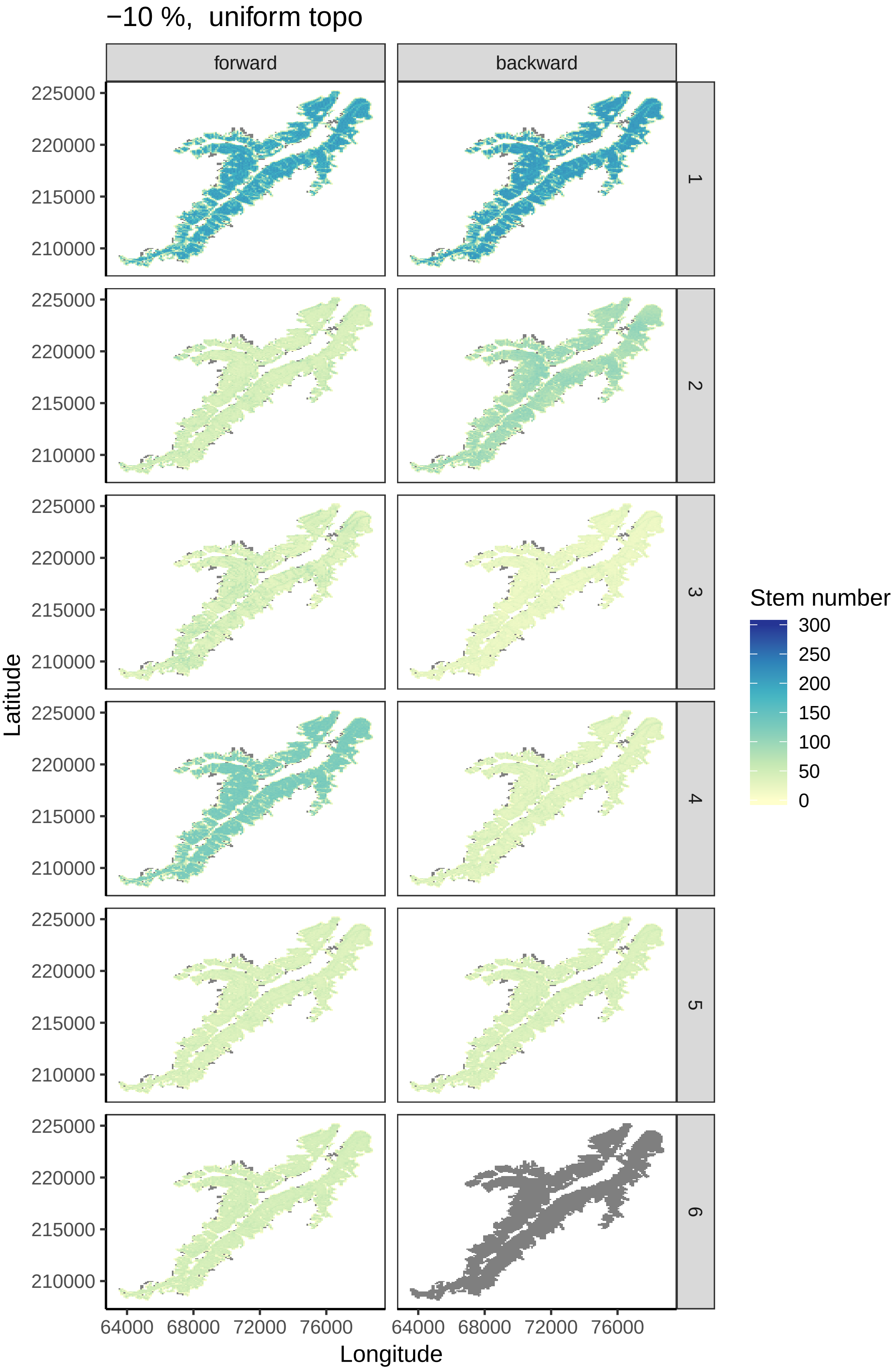
−10 %, uniform topo
forward
backward
1
2
3
4
5
6
225000
220000
215000
210000
Latitude
64000
68000
72000
76000
Longitude
Stem number
300
250
200
150
100
50
0

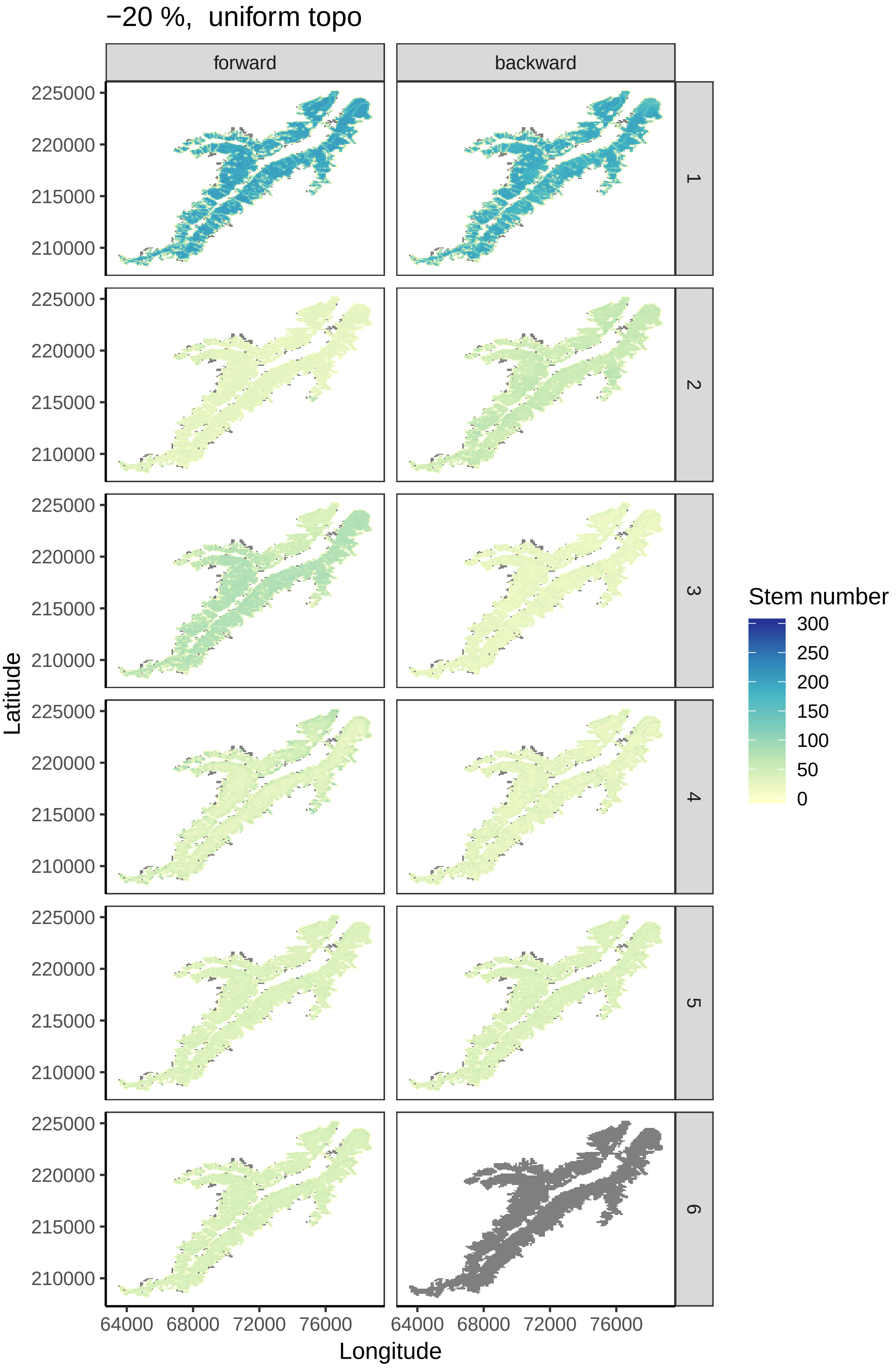
−20 %, uniform topo
forward
backward
1
2
3
4
5
6
225000
220000
215000
210000
Latitude
64000
68000
72000
76000
Longitude
Stem number
300
250
200
150
100
50
0

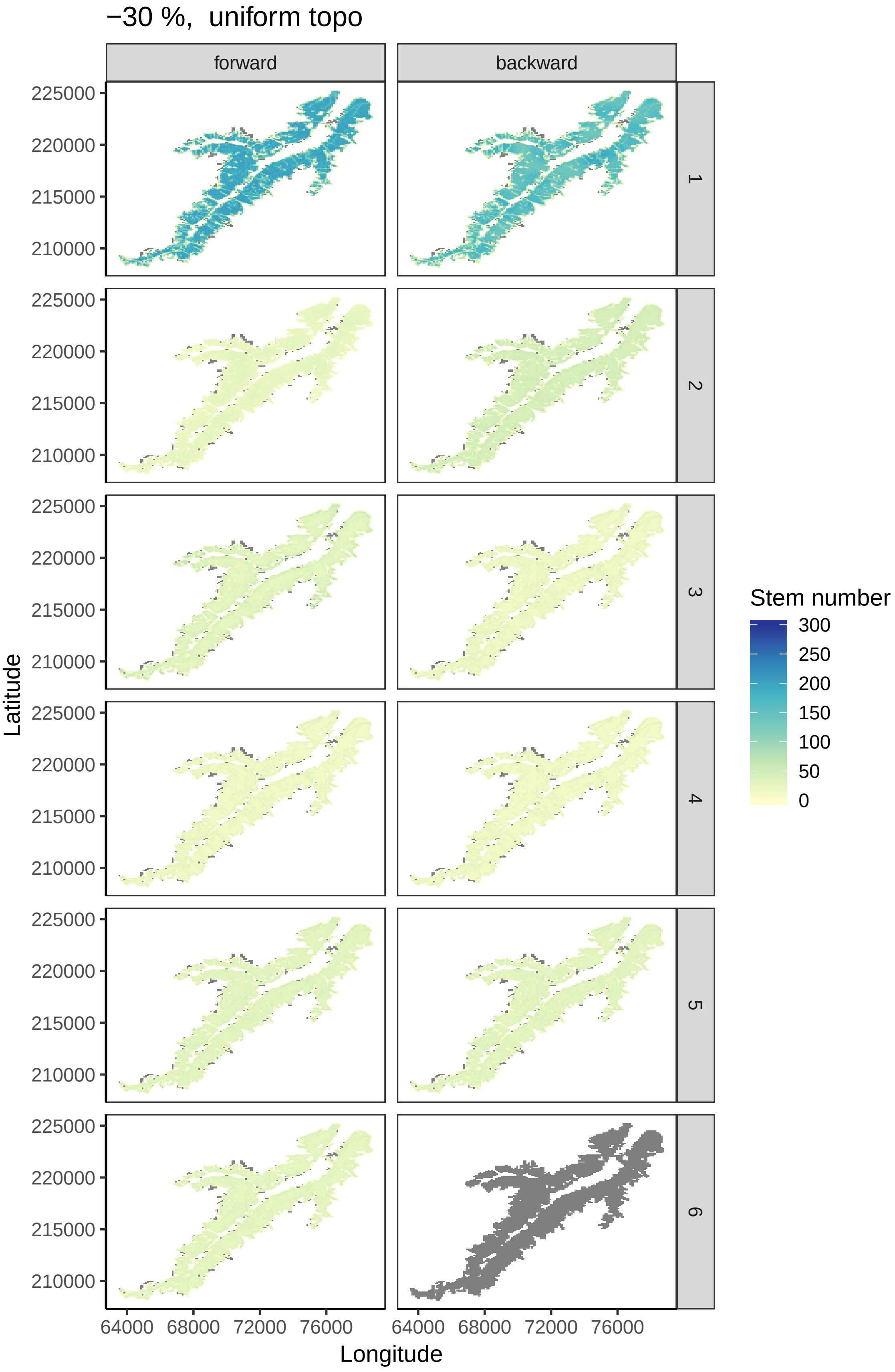

−30 %, uniform topo
forward
backward
1
2
3
4
5
6
225000
220000
215000
210000
Latitude
64000
68000
72000
76000
Longitude
Stem number
300
250
200
150
100
50
0

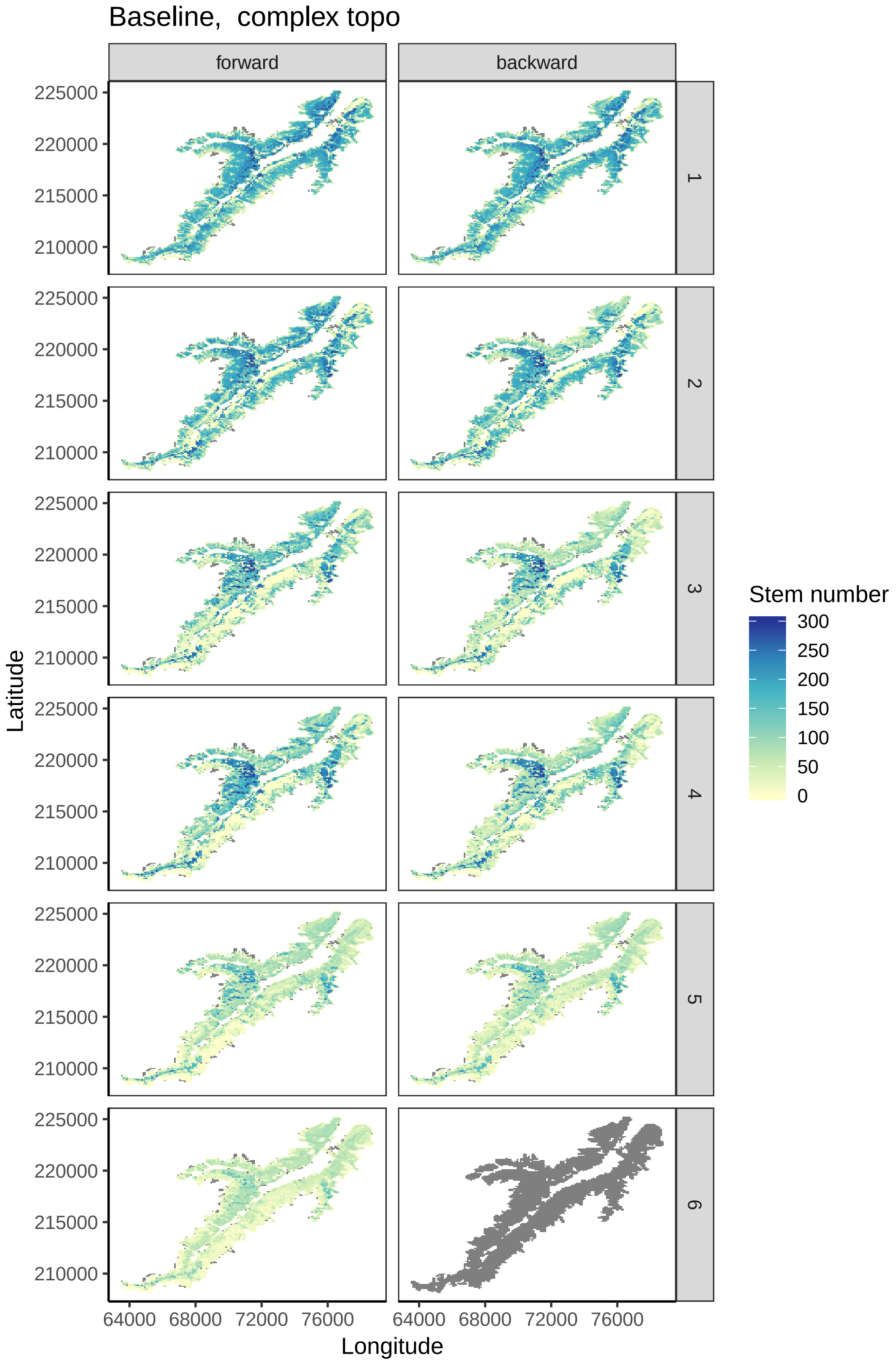

Baseline, complex topo
forward
backward
1
2
3
4
5
6
225000
220000
215000
210000
Latitude
64000
68000
72000
76000
Longitude
Stem number
300
250
200
150
100
50
0

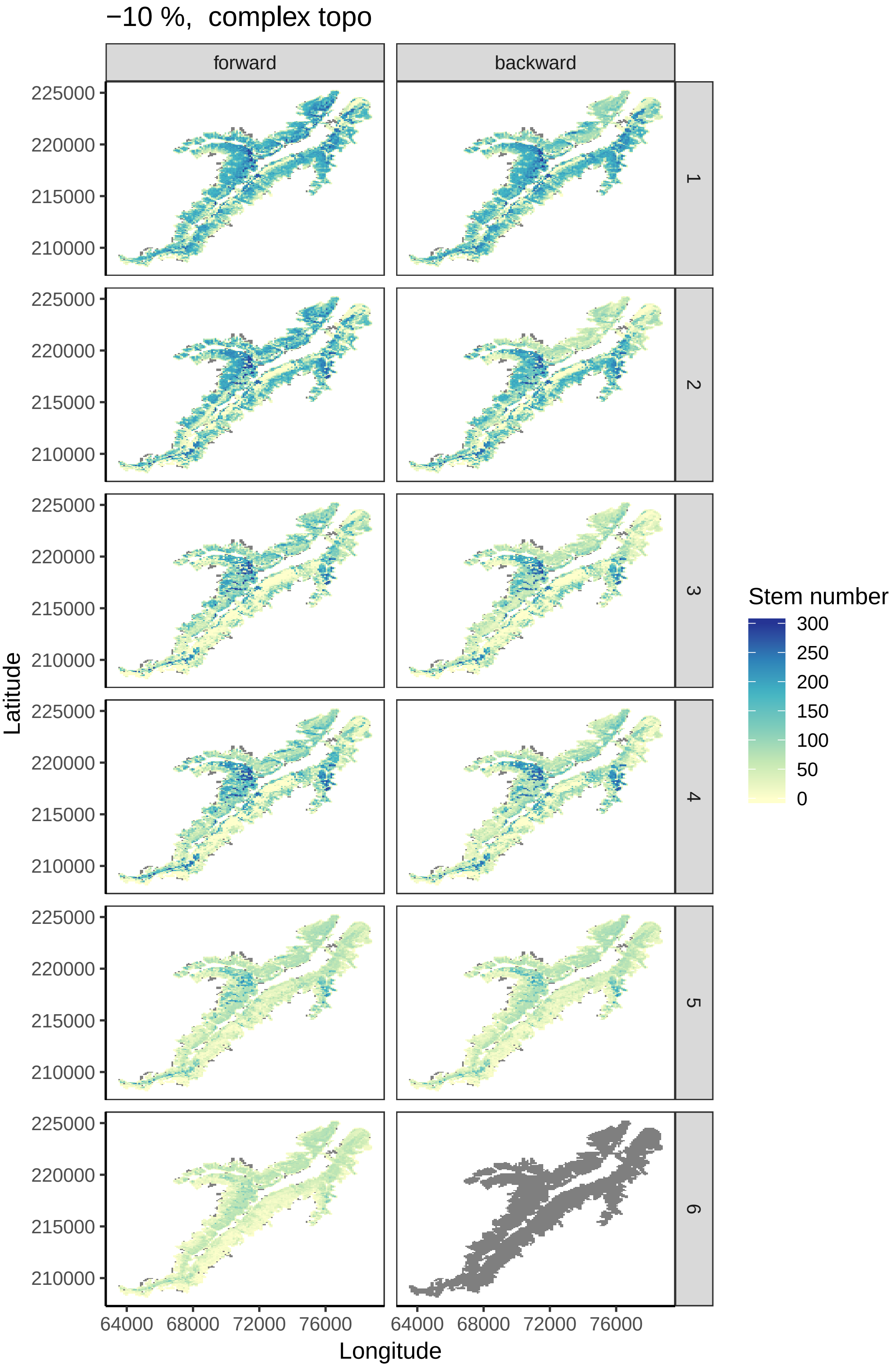

−10 %, complex topo
forward
backward
1
2
3
4
5
6
225000
220000
215000
210000
Latitude
64000
68000
72000
76000
Longitude
Stem number
300
250
200
150
100
50
0

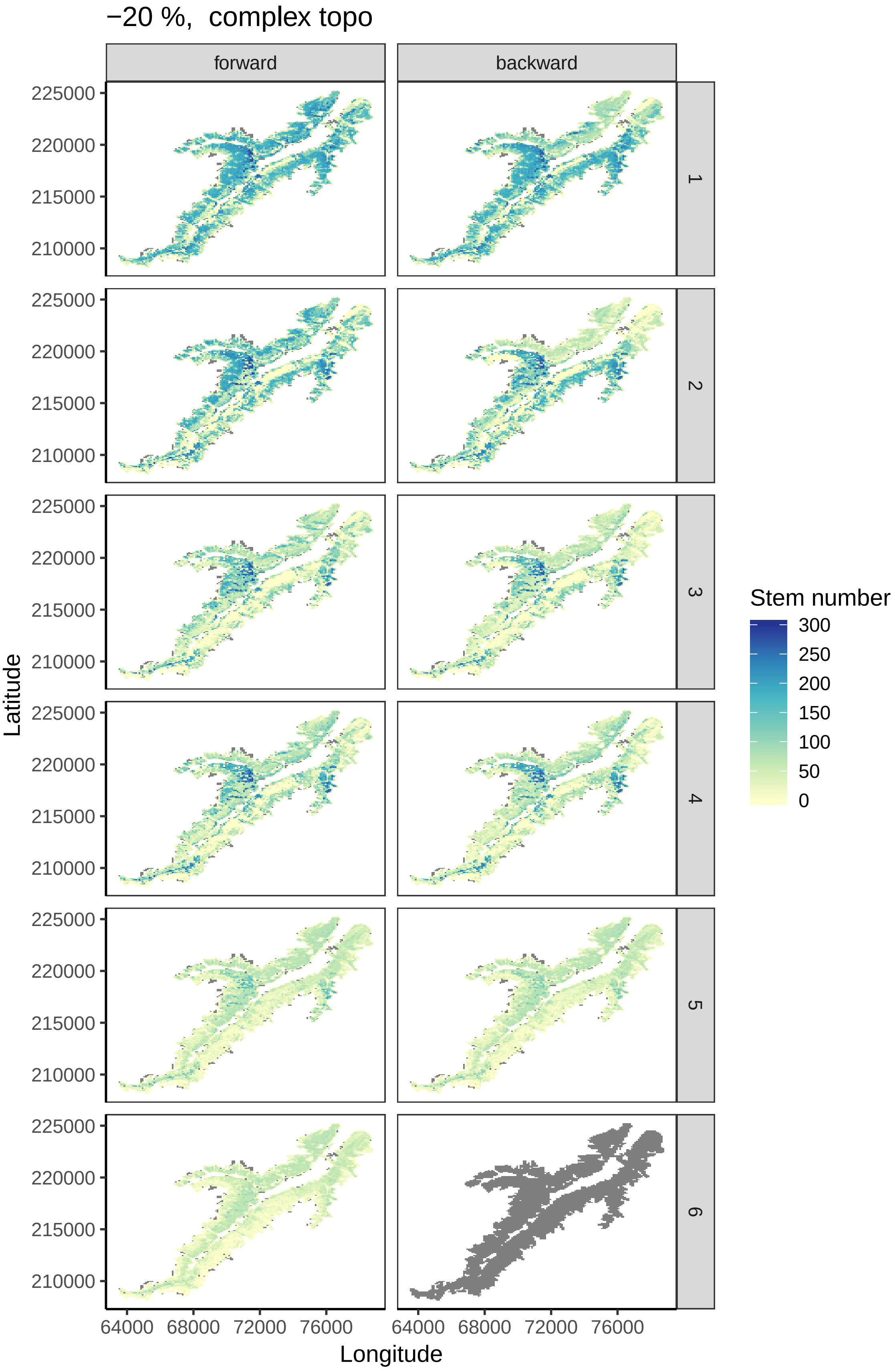
−20 %, complex topo
forward
backward
1
2
3
4
5
6
225000
220000
215000
210000
Latitude
64000
68000
72000
76000
Longitude
Stem number
300
250
200
150
100
50
0

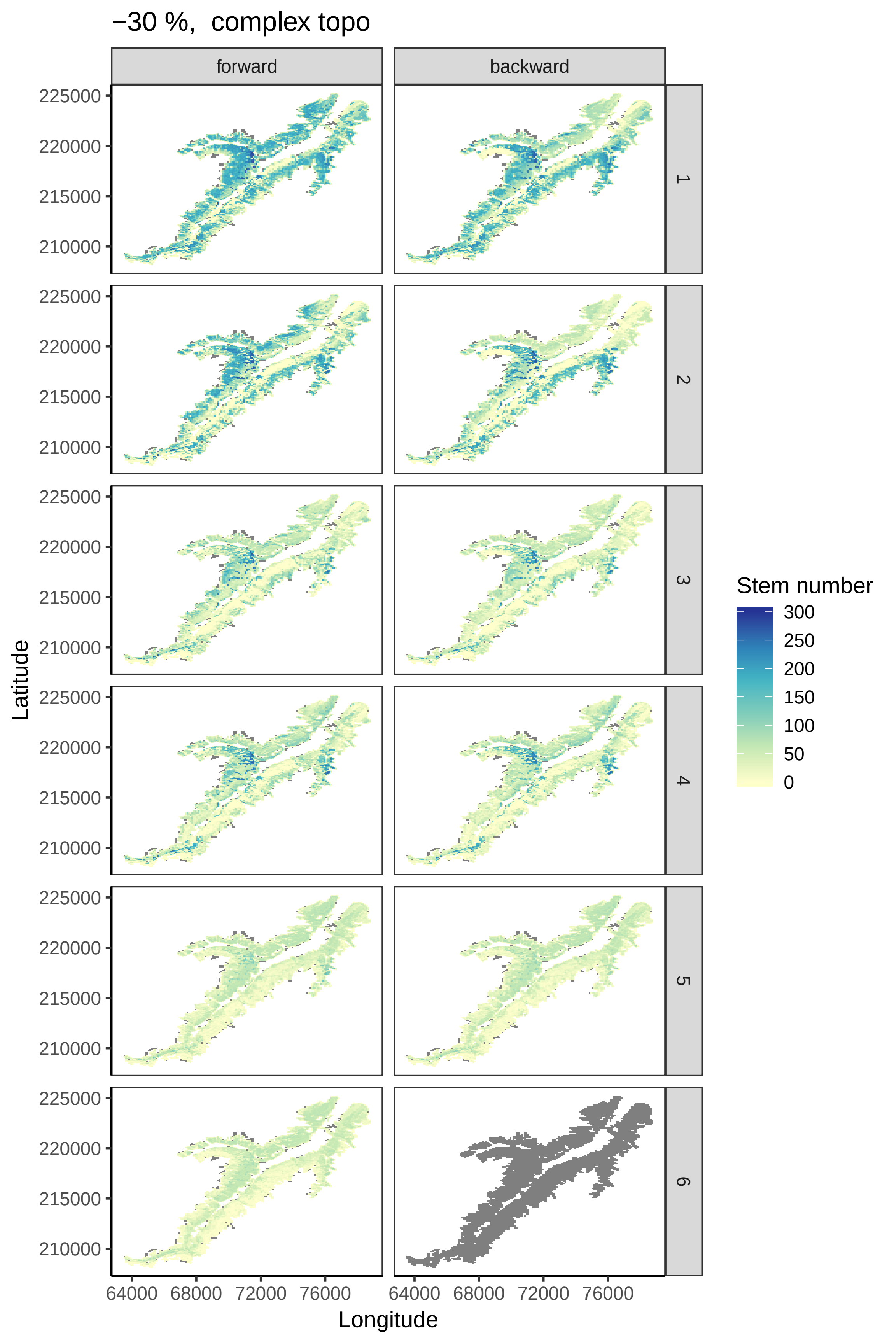

−30 %, complex topo
forward
backward
1
2
3
4
5
6
Latitude
225000
220000
215000
210000
Longitude
64000
68000
72000
76000
Stem number
300
250
200
150
100
50
0
